# A New Solution to the Grain Boundary Grooving Problem in Polycrystalline Thin Films When Evaporation and Diffusion Meet in Power Electronic Devices

**DOI:** 10.3390/mi15060700

**Published:** 2024-05-25

**Authors:** Tayssir Hamieh, Ali Ibrahim, Zoubir Khatir

**Affiliations:** 1 Faculty of Science and Engineering, Maastricht University, P.O. Box 616, 6200 MD Maastricht, The Netherlands; 2 Systèmes et Applications des Technologies de l’Information et de l’Energie (SATIE), Gustave Eiffel University, 25 Allée des Marronniers, 78000 Versailles, France; ali.ibrahim@univ-eiffel.fr (A.I.); zoubir.khatir@univ-eiffel.fr (Z.K.)

**Keywords:** mathematical solution, evaporation–condensation, diffusion, groove formation, degradation, thermal fatigue, electronic devices

## Abstract

This paper constituted an extension of two previous studies concerning the mathematical development of the grain boundary grooving in polycrystalline thin films in the cases of evaporation/condensation and diffusion taken separately. The thermal grooving processes are deeply controlled by the various mass transfer mechanisms of evaporation–condensation, surface diffusion, lattice diffusion, and grain boundary diffusion. This study proposed a new original analytical solution to the mathematical problem governing the grain groove profile in the case of simultaneous effects of evaporation–condensation and diffusion in polycrystalline thin films by resolving the corresponding fourth-order partial differential equation $\frac{\partial y}{\partial t}=C\frac{\partial^{2}y}{\partial x^{2}}-B\frac{\partial^{4}y}{\partial x^{4}}$ obtained from the approximation ${\left(\frac{\partial y}{\partial x}\right)}^{2}\ll 1$. The comparison of the new solution to that of diffusion alone proved an important effect of the coupling of evaporation and diffusion on the geometric characteristics of the groove profile. A second analytical solution based on the series development was also proposed. It was proved that changes in the boundary conditions of the grain grooving profile largely affected the different geometric characteristics of the groove profile.

## 1. Introduction

Power semiconductor devices and modules are critical components in various applications, including electric vehicles, renewable energy systems, and industrial automation. These devices often employ thin film technologies to achieve high performance and compact designs [1,2,3]. However, their reliability can be compromised over time due to fatigue phenomena, particularly in environments characterized by high temperatures and cyclic loading. Understanding fatigue mechanisms in thin films within the context of power semiconductor devices is essential for ensuring their long-term stability and efficiency [4,5,6].

In power semiconductor devices and modules, such as insulated gate bipolar transistors (IGBTs) and metal-oxide-semiconductor field-effect transistors (MOSFETs), thermal stress arises from the operation and dissipation of heat during switching events and continuous operation [7,8,9,10,11,12]. The cyclic nature of these temperature fluctuations subjects thin films within the devices to mechanical strain, leading to fatigue-related phenomena [13]. Groove formation, a characteristic manifestation of thermal stress, can occur on the surfaces of critical components such as substrates, interconnects, and passivation layers [14,15,16]. These grooves typically emerge along regions subjected to maximum thermal stress, such as the interfaces between different material layers or near localized heat sources. Over time, the repetitive stress cycles deepen and widen these grooves, potentially compromising the electrical and thermal performance of the semiconductor device. Additionally, the presence of grooves can increase the risk of electrical breakdown and thermal hotspots, further exacerbating reliability issues [17,18].

**Figure 1:**
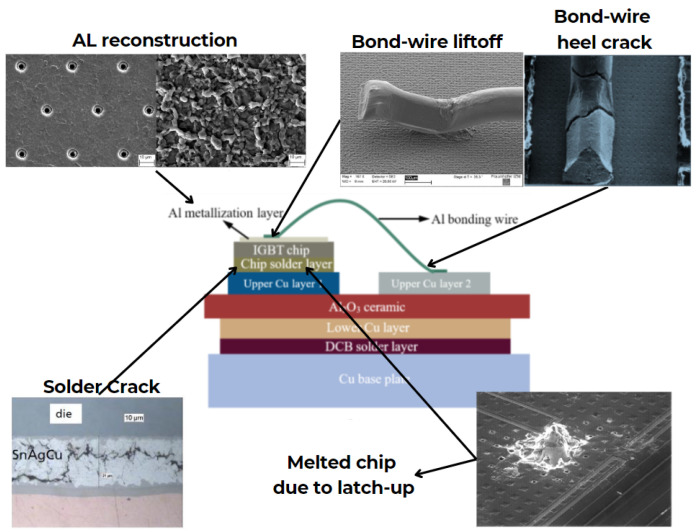
Failure mechanisms in IGBT modules.

IGBT power modules are intricately structured with multiple material layers, subject to diverse sources of stress that can induce gradual degradation through intrinsic (chip-related) and extrinsic (package-related) failure mechanisms. Figure 1 provides illustrations of several of these mechanisms. However, the predominant causes of degradation primarily stem from thermomechanical factors, owing to temperature fluctuations experienced during the module’s operational lifespan. These fluctuations lead to progressive deterioration between successive layers, particularly pronounced in layers exhibiting significant mismatches in the coefficient of thermal expansion (CTE). Additionally, the degradation of metallization and the interface between metallization and bond wires represent prominent contributors to power module failures, further underscoring the multifaceted nature of degradation processes in these modules.

In the context of power semiconductor devices, groove formation due to thermal stress is intricately linked to evaporation and diffusion phenomena. Elevated temperatures within the device can accelerate the evaporation of volatile species from thin film materials or encapsulants, altering their composition and mechanical properties [19,20]. This loss of material may exacerbate stress concentrations and promote the initiation of grooves along vulnerable regions of the device [21,22,23,24]. Furthermore, diffusion processes play a crucial role in redistributing material within the thin films of power semiconductor devices. Diffusion-induced phenomena, such as interdiffusion at material interfaces or dopant migration within semiconductor layers, can influence the mechanical and electrical properties of the device [25,26]. The interplay between diffusion and thermal stress contributes to the evolution of grooves and the overall degradation of device performance over time [27,28,29].

Fatigue in thin films within power semiconductor devices and modules poses significant challenges to their reliability and performance under demanding operating conditions [30,31]. The formation of grooves due to thermal stress, coupled with evaporation and diffusion phenomena, represents complex interdependencies that must be addressed to enhance device durability [31,32,33,34,35].

The problem of grain boundary grooving in polycrystalline thin films was studied by many researchers [14,15,16,36,37,38,39,40,41,42,43,44,45,46,47,48,49]. The works of Mullins [14,15,16] were devoted to solving the problem governing the profile of grain boundary grooving. The mathematical formulation of this problem was developed by several scientists by focusing on the evaporation–condensation and surface diffusion by adopting several non-linear methods [37,38,39,40,41,42,43,44,45,46,47,48] due to the non-linear partial differential equation previously formulated by Mullins [14,15,16,50]. 

Li et al. [51] studied the interaction between grain boundary and cascade in some composites with reference to single-element Ni metal by applying molecular dynamics. They showed that the capture of point defects by grain boundary was effective, and the number of captured defects was related to the distance between primary knock-on atoms and grain boundary.

Chepak-Gizbrekht and Knyazeva [52] investigated a two-dimensional model of grain-boundary diffusion with consideration for triple junctions. They studied the effect of grain size on diffusion in a polycrystal under varying temperature conditions; found that the diffusant behaved non-monotonously, accumulating and consuming at the triple junction during diffusion; and showed a dependency of the diffusion coefficient on the grain size.

Moshtaghi et al. [53] studied the effect of ferrite grain boundary in some materials on hydrogen trapping, desorption, diffusion, and grain size. Their modeling results showed that in grain sizes smaller than a certain critical grain size, the hydrogen diffusion coefficient decreases with decreasing grain size, indicating that H trapping dominates short-circuit diffusion mechanisms along high-angle grain boundaries (HAGBs). They suggested that the fraction of acicular ferrite grains can define the HAGB content in the alloy and can be a determinant factor in the behavior of weldments in H-containing media. 

Schweizer et al. [54] studied the volume and grain boundary diffusion processes of tungsten atoms in a metal matrix on the atomic scale. Using in situ high-resolution scanning transmission electron microscopy, they followed the random movement of single atoms within a lattice at elevated temperatures. They used the STEM to confirm random walk processes, quantify diffusion kinetics, and distinctly separate diffusion in the volume from diffusion along defects. They also confirmed that the mechanism of grain boundary diffusion can rely on both lattice sites and excess volume depending on the type of boundary.

Wang et al. [55] modified the microstructure of the grain boundary phase through the diffusion method, improved the coercivity of magnetic powders, and then their thermal stability. They studied the effect of GBD on the microstructures and magnetic properties of some magnetic powders and the modification of different content of the alloy to grain boundary and microstructure.

Zhao et al. [56] used a grain growth model based on a Monte Carlo algorithm integrated with the diffusion model by considering grain growth and diffusion in nanocrystalline materials. They studied the effects of grain growth and impurity on diffusion and provided a numerical strategy to study microstructure evolution in nanocrystalline materials.

Chatzimichail et al. [57] determined the temperature dependence of the surface energy and the grain boundary energy of polycrystalline MgO, as well as of the interfacial energy of MgO in contact with liquid Ag and Cu. They studied the mechanism and the kinetics of mass transport in the grooving process at the grain boundaries/free surface intersection of MgO. They found that surface diffusion as a mass transport mechanism is responsible for grain boundary grooving in polycrystalline MgO.

The kinetics of atom adsorption, desorption, and diffusion in polycrystalline materials were analyzed [58] to understand the influence of grain boundaries and grain size. The effect of the ratio of grain boundary and grain diffusion coefficients in concentration profiles was studied, as well as the influence of grain size on diffusion processes and the impact of kinetic processes taking place on the surface.

Gheno et al. [59] studied the grain boundary diffusion of chromium in polycrystalline nickel by SIMS and examined the shape of individual grain boundary profiles, and the applicability of the existing methods to process grain boundary diffusion profiles.

By advancing our understanding of these mechanisms and developing robust materials, designs, and manufacturing processes, it is possible to mitigate the effects of fatigue and improve the reliability of power semiconductor devices. Through interdisciplinary research and innovation, we can ensure the continued advancement and widespread adoption of power electronics technology in critical applications. Peyghami et al. [60] proposed a long-term performance indicator for power electronic converters based on their reliability. The converter reliability was represented by constant lifetime curves, which have been developed using an Artificial Neural Network (ANN) under different operating conditions. These proposed lifetime curves presented the long-term performance of converters facilitating optimal system-level design for reliability, reliable operation, and maintenance planning in power electronic systems.

We proposed, in previous works [61,62,63], analytical solutions to the mathematical problem in the case of the evaporation–condensation in polycrystalline thin films by resolving the corresponding second non-linear partial differential equation [61,62] without any approximation when materials are submitted to thermal and mechanical stress and fatigue effects. One proved the non-validity of Mullins approximation that neglected the first derivative in the mathematical equation associated with the evaporation case. In a recent study, we studied the problem of diffusion in thin polycrystalline films, resolved the fourth-order partial differential equation of diffusion, and provided an analytical solution to the equation $\frac{\partial y}{\partial t}+B\frac{\partial^{4}y}{\partial x^{4}}=0$, obtained by admitting the approximation ${y\prime }^{2}\ll 1$. However, we did not find any analytical study of the association of evaporation and diffusion phenomena in polycrystalline films.

In this paper, we studied the effect of the simultaneous combination of evaporation–condensation and diffusion on the grain groove profile by resolving the fourth-order partial differential equation associated with these two combined effects. An analytical solution was proposed and detailed in this present work and compared to the solution obtained separately in the diffusion and evaporation–condensation cases.

## 2. Mathematical Equation of the Grain Boundary Grooving

The differential equation describing the grain boundary grooving when evaporation and diffusion simultaneously act in polycrystalline thin films is given by Equation (1) or (2):

(1) $$\frac{\partial y}{\partial t}=C\frac{\frac{\partial^{2}y}{\partial x^{2}}}{\left\lfloor 1+{\left(\frac{\partial y}{\partial x}\right)}^{2}\right\rfloor }-B\;\frac{\partial }{\partial x}\left[{\left[1+{\left(\frac{\partial y}{\partial x}\right)}^{2}\right]}^{-1/2}\frac{\partial }{\partial x}\left[\frac{\frac{\partial^{2}y}{\partial x^{2}}}{{\left\lfloor 1+{\left(\frac{\partial y}{\partial x}\right)}^{2}\right\rfloor }^{3/2}}\right]\right]$$

or

(2) $$\frac{\partial y}{\partial t}=C\frac{y^{\prime \prime }}{\left(1+y^{\prime 2}\right)}-B\;\frac{\partial }{\partial x}\left[{\left(1+{y\prime }^{2}\right)}^{-1/2}\;\frac{\partial }{\partial x}\left[\frac{y^{\prime \prime }}{{\left(1+y^{\prime 2}\right)}^{3/2}}\right]\right]$$

where $y^{\prime }=\frac{\partial y}{\partial x}$ and $y^{\prime \prime }=\frac{\partial^{2}y}{\partial x^{2}}$ and the boundary conditions are given by Equation (3):

(3) $$\left\{\begin{array}{c} y\left(x,\;0\right)=0\\ y\prime \left(0,\;t\right)=\tan\theta =m\\ \underset{x\to \infty }{\lim}y^{\prime }\left(x,\;t\right)=0\\ \underset{x\to \infty }{\lim}y^{\prime \prime }\left(x,\;t\right)=0\\ y^{\prime \prime \prime }\left(0,\;t\right)=0 \end{array}\right.$$

where x and y(x,t) represent the coordinates of a point at the surface along the axis normal to the initial flat surface at a time t. C and B are two constants relative, respectively, to evaporation and diffusion phenomena. C and B are given by Equations (4) and (5):

(4) $$C\left(T\right)=\frac{P_{0}\left(T\right)\;\gamma \left(T\right)\;{N\omega }^{2}}{\sqrt{2\pi MRT}}$$

(5) $$B(T)=\frac{D_{s}\gamma {N\omega }^{2}N_{S}}{RT}$$

where γ is the isotropic surface energy or tension of the metal/vapor interface, $P_{0}\left(T\right)\;$is the vapor pressure at temperature T in equilibrium with the plane surface of the metal, ω is the atomic volume, M is the molar mass of the metal, R is the perfect gas constant, $D_{s}$ is the surface diffusivity, and $N_{S}$ is the number of diffusing atoms per unit area.

Equation (2) can be written as follows:

(6) $$\frac{\partial y}{\partial t}=C\frac{y^{\prime \prime }}{\left(1+y^{\prime 2}\right)}-B\;\left[\frac{y^{\prime \prime \prime \prime }{\left(1+y^{\prime 2}\right)}^{2}-\left(y^{\prime \prime 3}+{10y}^{\prime }y^{\prime \prime }y^{\prime \prime \prime }\right)\left(1+y^{\prime 2}\right)+18y^{\prime 2}y^{\prime \prime 3}}{{\left(1+y^{\prime 2}\right)}^{4}\;}\right]$$

By taking the following variable changes Equation (7):

(7) $$\left\{\begin{array}{c} u\left(x,\;t\right)=\frac{x}{{\left(Bt\right)}^{1/4\;}}\\ y\left(x,\;t\right)=m\;{\left(Bt\right)}^{1/4\;}g\left[\frac{x}{{\left(Bt\right)}^{1/4\;}}\right]\\ y\left(u,\;t\right)=m\;{\left(Bt\right)}^{1/4\;}g\left(u\right) \end{array}\right.$$

The different derivatives of $y\left(x,\;t\right)$ and $u\left(x,\;t\right)$ are given by Equation (8):

(8) $$\left\{\begin{array}{c} \frac{\partial u}{\partial x}=\frac{1}{{\left(Bt\right)}^{1/4\;}}\\ \frac{\partial u}{\partial t}=-\frac{u}{4t}\\ \frac{\partial y}{\partial t}=\frac{1}{4}\frac{mB}{{\left(Bt\right)}^{3/4\;}}\;\left(g\left(u\right)-u\frac{\partial g}{\partial u}\right)\\ y^{\prime }=m\;\frac{\partial g}{\partial u}\\ y^{\prime \prime }=\frac{m}{{\left(Bt\right)}^{1/4\;}}\;\frac{\partial^{2}g}{\partial u^{2}}\\ y^{\prime \prime \prime }=\frac{m}{{\left(Bt\right)}^{2/4\;}}\;\frac{\partial^{3}g}{\partial u^{3}}\\ y^{\prime \prime \prime \prime }=\frac{m}{{\left(Bt\right)}^{3/4\;}}\;\frac{\partial^{4}g}{\partial u^{4}} \end{array}\right.$$

One deduced the following general equation of the grain groove profile:

$$\frac{1}{4}\;\left(g\left(u\right)-u\frac{\partial g}{\partial u}\right)=C{\left(\frac{t}{B}\right)}^{1/2}\frac{\;\frac{\partial^{2}g}{\partial u^{2}}}{\left(1+{m\left(\frac{\partial g}{\partial u}\right)}^{2}\right)}-\frac{\;\frac{\partial^{4}g}{\partial u^{4}}{\left(1+{m\left(\frac{\partial g}{\partial u}\right)}^{2}\right)}^{2}-m^{2}\left(\;{\left(\frac{\partial^{2}g}{\partial u^{2}}\right)}^{3}+10\;\frac{\partial g}{\partial u}\;\frac{\partial^{2}g}{\partial u^{2}}\;\frac{\partial^{3}g}{\partial u^{3}}\right)\left(1+{m\left(\frac{\partial g}{\partial u}\right)}^{2}\right)+18m^{4}\;{\left(\frac{\partial g}{\partial u}\right)}^{2}{\left(\frac{\partial^{2}g}{\partial u^{2}}\right)}^{3}}{{\left(1+{m\left(\frac{\partial g}{\partial u}\right)}^{2}\right)}^{4}\;}$$

The above partial differential equation cannot analytically be resolved without approximation. In the following sections, we resumed the essential results obtained in previous studies [61,62,63].

## 3. Case of Evaporation/Condensation [61,62]

The evaporation–condensation problem is governed by the following mathematical equation:

(9) $$\frac{\partial y}{\partial t}=C(T)\frac{y\prime \prime \;\left(x\right)}{\left(1+{y\prime \;\left(x\right)}^{2}\right)}$$

Equation (9) can be transformed with the same notations to the following equation:

(10) $$y\prime \prime \left(u\right)+2u\;\frac{1}{4Ct}{y\prime \left(u\right)}^{3}+2uy\prime \left(u\right)=0$$

or

(11) $$y\prime \prime \left(u\right)=-2uy\prime \left(u\right)\;\left[1+\frac{1}{4Ct}{y\prime \left(u\right)}^{2}\right]$$

To resolve the non-linear differential Equation (11), Mullins did the approximation of a small slope by supposing $\left|y\prime \right|\ll 1$. He wrote the following:

(12) $$y\prime \prime \left(u\right)=-2uy\prime \left(u\right)$$

The integration of Equation (12) gave the following:

$$y\prime \left(u\right)=Ae^{-u^{2}}$$

With *A* being a constant of the problem determined by the condition boundary $y\prime \left(0,\;t\right)=m$

$$A=2m\sqrt{Ct}$$

The solution of the differential Equation (12) obtained by the approximated Mullins problem is given by Equation (13):

(13) $$y\left(x,\;t\right)=-m\sqrt{\pi Ct}\;\left[1-\frac{2}{\sqrt{\pi }}\int_{0}^{\frac{x}{2\sqrt{Ct}}}e^{-u^{2}}du\right]$$

In previous studies [61,62], we corrected the solution given by Mullins by considering general Equation (10) without any approximation and obtained the following equation:

(14) $$y\left(x,\;t\right)=\int_{\infty }^{x/2\sqrt{Ct}}\;\frac{\;sin\;\theta }{\sqrt{e^{v^{2}/(2Ct)}-{sin}^{2}\theta }}\;dv$$

And the final solution is given by Equation (15):

(15) $$y\left(x,\;t\right)=-\sqrt{\pi Ct}\;sin\;\theta [erfc\left(\frac{x}{2\sqrt{Ct}}\right)+\overset{\infty }{\underset{n=1}{\sum }}\frac{\left(2n\right)!}{{\left(n!\right)}^{2}2^{2n}\;\sqrt{3n}}{\;sin}^{2n}\theta \;(erfc\left(\;\frac{x\sqrt{3n}}{2\sqrt{Ct}}\right))]$$

where θ is the groove contact angle.

## 4. Diffusion Case [63]

The diffusion case previously studied [63] was relative to the mathematical solution of the formation of grain boundary grooving in polycrystalline thin films by taking the approximation ${y\prime }^{2}\ll 1$. The following fourth differential equation (Equation (16)) was obtained:

(16) $$g^{\prime \prime \prime \prime }-\frac{1}{4}ug^{\prime }+\frac{1}{4}g=0$$

Equation (16) satisfied the previous boundary conditions.

The analytical solution of Equation (16) was obtained and given by Equation (17):

(17) $$g\left(u\right)=\left\{\begin{array}{c} g_{1}\left(u\right)\;\;\;\;\;\;\;\;\;\;\;for\;u\leq \frac{2^{5/2}}{3^{3/4}}\;\\ g_{2}\left(u\right)\;\;\;\;\;\;\;\;\;\;for\;u\geq \frac{2^{5/2}}{3^{3/4}} \end{array}\right.$$

where the explicit expressions $g_{1}\left(u\right)$ and $g_{2}\left(u\right)$ of the solution were given as follows:

(18) $$\left\{\begin{array}{c} g_{1}\left(u\right)=e^{-\sqrt{\frac{\lambda_{1}}{2}}\;u}\;\left(A_{11}cos\;\left(\sqrt{\frac{u}{8\sqrt{2\lambda_{1}}}+\frac{\lambda_{1}}{2}}\;u\right)+A_{21}sin\;\left(\sqrt{\frac{u}{8\sqrt{2\lambda_{1}}}+\frac{\lambda_{1}}{2}}\;u\right)\right)\;\\ g_{2}\left(u\right)=e^{-\sqrt{\frac{\lambda_{2}}{2}}\;u}\left(A_{12}cos\left(\sqrt{\frac{u}{8\sqrt{2\lambda_{2}}}+\frac{\lambda_{2}}{2}}\;u\right)+A_{22}sin\left(\sqrt{\frac{u}{8\sqrt{2\lambda_{2}}}+\frac{\lambda_{2}}{2}}\;u\right)\right)\; \end{array}\right.$$

It was proved that the solution given by Hamieh et al. [63] revealed a damped sinusoidal groove profile in the case of electronic power devices. The expressions of zeros, minima, and maxima of the profile as a function of the order number, as well as detailed information about the groove profile y(x) and its derivatives, were given. The comparison of this new solution with Mullins’ results showed that the approximated solution obtained by Mullins [14,15,16] overestimated the values of the groove geometric characteristics, exceeding the actual values by more than 2.5 times. Additionally, valuable insights into the diffusion behavior of various metals were gained through this study. The new expressions relative to the diffusion were used to study several metals such as *Cu, Al, Sr, Li, Cs, Ti, Co, Ga*, and *Tl* and give the geometric parameters such as the depth $h_{Max}$ and the width $w_{Max}$ of the grain groove.

## 5. Study of the Combination of Evaporation/Condensation and Diffusion Cases 

The evaporation/condensation and diffusion phenomena in polycrystalline thin films were studied separately in the literature. We did not find a complete and rigorous development of the simultaneous combination of evaporation/condensation and diffusion. In this section, we developed a new mathematical solution to this case by supposing that ${y\prime }^{2}\ll 1$.

In this case, Equation (6) can be written as

(19) $$\frac{\partial y}{\partial t}=Cy^{\prime \prime }-By^{\prime \prime \prime \prime }$$

By using the reduced function g(u), Equation (19) became as

(20) $$g^{\prime \prime \prime \prime }-\frac{C}{B}\;{\left(Bt\right)}^{1/2\;}g^{\prime \prime }-\frac{1}{4}\;ug^{\prime }+\frac{1}{4}g=0$$

The ratio $\frac{C}{B}$ is given by

(21) $$\frac{C}{B}=\frac{\mu \;P_{0}\;\;}{D_{s}N_{S}}\;\sqrt{\frac{RT}{2\pi M}}$$

where *M* is the molar mass of the metal and *R* is the perfect gas constant.

## 6. Mathematical Resolution of the Combined Cases

Equation (21) can be resolved by using the following characteristic equation:

(22) $$r^{4}-a{\;r}^{2}-\frac{1}{4}u\;r+\frac{1}{4}=0$$

The thermo-diffusion coefficient a is given by Equation (23):

(23) $$a=C{\left(\frac{t}{B}\right)}^{1/2}$$

Let us consider the following equation valid for all values of *μ*:

(24) $${r^{4}-a{\;r}^{2}-\frac{1}{4}u\;r+\frac{1}{4}=\left(r^{2}+\mu \right)}^{2}-\frac{{\left(8\mu +4a\right)r}^{2}+u\;r+{4\mu }^{2}-1}{4}$$

Our method consisted of transforming the expression *E* into a perfect square by finding the double root of this second-degree equation. *E* is given by Equation (25):

(25) $$E={\left(8\mu +4a\right)r}^{2}+u\;r+{4\mu }^{2}-1$$

This led to the study of the discriminant ∆ of Equation (24) by distinguishing two cases relative to the positive and negative signs of $\left(u-\sqrt{a_{1}+a_{2}}\right)$ or $\left(u-u_{0}\right)$ where, $u_{0}$, $a_{1}$, and $a_{2}$ are given by Equation (26):

(26) $$\left\{\begin{array}{c} u_{0}=\sqrt{a_{1}+a_{2}}\\ a_{1}=\frac{2^{5}\;\left(a^{3}-9a\right)}{3^{3}}\\ a_{2}={\frac{2^{5}}{3^{3}}\left(a^{2}+3\right)}^{3/2} \end{array}\right.$$

The analytical solution $g\left(u\right)$ was obtained by applying the boundary conditions to the studied cases, thus allowing the study of the profile variation of the grain groove (all mathematical details are given in Appendix A).

The final analytical solution of the grooving profile in the case of the combined cases of evaporation and diffusion is given as follows:

(27) $$g\left(u\right)=\left\{\begin{array}{c} g_{2}\left(u\right)\;\;\;\;\;\;\;\;\;\;\;for\;u\leq u_{0}\;\\ g_{1}\left(u\right)\;\;\;\;\;\;\;\;\;\;for\;u\geq u_{0} \end{array}\right.$$

where the functions $g_{1}\left(u\right)$ and $g_{2}\left(u\right)$ are given, respectively, by Equations (28) and (29):

(28) $$g_{1}\left(u\right)=Exp\left[\left(-\rho^{1/2}\;con\left(\frac{\theta }{2}\right)\right)u\right]\left[A_{11}cos\left[\left(\sqrt{\left(-2\mu -a\right)}-sin\left(\frac{\theta }{2}\right)\right)\;u\right]+A_{21}sin\left[\left(\sqrt{\left(-2\mu -a\right)}-sin\left(\frac{\theta }{2}\right)\right)\;u\right]\right]$$

$$g_{2}\left(u\right)=e^{-\frac{1}{2}\times \sqrt{\left(2\mu +a\right)}\;u}\left(A_{12}Exp\left(\frac{1}{2}\times \sqrt{\left(a-2\mu \right)-\frac{u}{2\sqrt{\left(a+2\mu \right)}}}\;u\right)+A_{22}Exp\left(-\frac{1}{2}\times \sqrt{\left(a-2\mu \right)-\frac{u}{2\sqrt{\left(a+2\mu \right)}}}\;u\right)\right)$$

(29) $$+e^{\frac{1}{2}\times \sqrt{\left(2\mu +a\right)}\;u}\left(A_{32}Exp\left(\frac{1}{2}\times \sqrt{\left(a-2\mu \right)+\frac{u}{2\sqrt{\left(a+2\mu \right)}}}\;u\right)+A_{42}Exp\left(-\frac{1}{2}\times \sqrt{\left(a-2\mu \right)+\frac{u}{2\sqrt{\left(a+2\mu \right)}}}\;u\right)\right)$$

The various expressions of the variables μ(u), θ(u), ρ(u) and of the problem constants ($A_{11},\;A_{21},\;A_{12},\;A_{22},\;A_{32},\;A_{42})\;$are provided in Appendix A.

**Figure 2:**
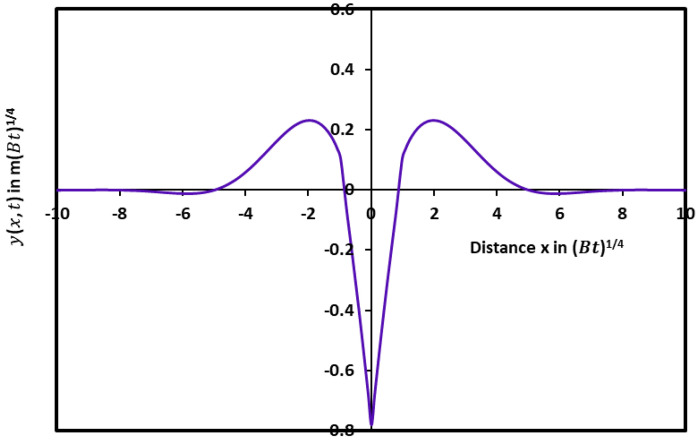
Groove profile giving $y\left(x,\;t\right)$ as a function of the distance x in the case of simultaneous effects of evaporation/condensation and diffusion.

The mathematical solution given by Equations (27)–(29) led to the drawing of the variations of the profile $y\left(x,\;t\right)$ in Figure 2 as a function of the distance *x* by taking the symmetric axis of the groove as the *y*-axis.

**Figure 3:**
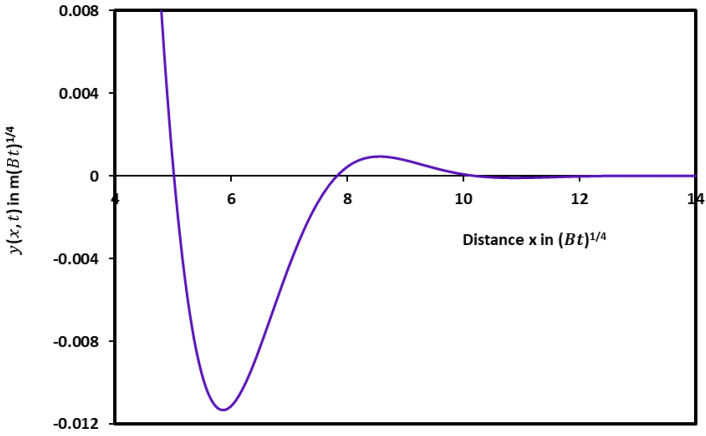
A part of damped sinusoidal profile of the groove profile giving $y\left(x,\;t\right)$ as a function of the distance x showing the first minimum and the second maximum of the profile.

The evolution of $y\left(x,\;t\right)$ in Figure 2 showed similar variations with the curve obtained with the diffusion case alone. A damped sinusoidal profile of the groove with an infinity of maxima, minima, and zeros of the solutions was revealed (Figure 3) with smaller oscillations. Figure 3 clearly shows the first minimum and the second maximum of the profile with a rapidly decreasing amplitude with the distance x.

The first values of the different parameters of the grain groove shape, such as the coordinates of maxima ($x_{Max};$ $y_{Max}$) and minima ($x_{min};$ $y_{min}$) of the function y(x,t), the differences between two consecutive maxima ($∆x_{Max}$) and two minima ($∆x_{min}$), and the zeros ($x_{0}$) of *y,* obtained from the solution $y\left(x,\;t\right)$ were presented in Table 1 for the first eight numbers by comparing the combined effect of evaporation and diffusion and the lone diffusion case.

**Figure 4:**
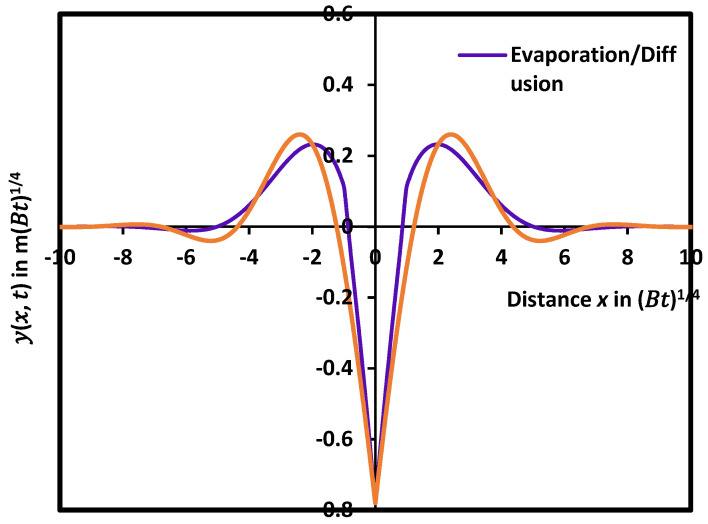
Comparison between the case of the lone diffusion and the combined evaporation and diffusion as a function of the distance from the symmetric axis of the groove.

Table 1 shows that the maximum $y_{Max}$ and the minimum $y_{min}$ of the solution y(x,t) decreased towards zero when *x* increased to infinity. However, the difference between the two maxima $∆x_{Max\;}$ or two minima $∆x_{min}$ of the grain groove profile decreased and tended to zero at infinity. The different parameters of the grain groove profile presented some differences in their values when considering the combination of evaporation and diffusion phenomena compared to the lone diffusion case (Table 1). The curves relative to the comparison between the case of the diffusion alone and the combined evaporation and diffusion as a function of the distance were plotted in Figure 4. The maximum $y_{Max}$ of the grain profile in the combination of evaporation and diffusion effects showed a decrease in its value with respect to the case of diffusion. The same decrease was observed for all maxima $y_{Max}$ and minima $y_{min}$ of the function $y\left(x,\;t\right)$. However, the separation distance between two consecutive maxima $d_{Max}$ or minima $d_{min}$ increased relatively to the diffusion case.

To clarify the difference in the diffusion process and the combined effects, one gave, in Table 2, the ratios of the different parameters in the two examined cases. The case of the diffusion alone was symbolized by the letter D, whereas the evaporation/condensation combined with the diffusion was represented by ECD. The results in Table 2 showed that the values of $x_{Max\;}$, $x_{min}$, $∆x_{Max\;}$, and $x_{min}$ are practically the same in ECD and D. However, an important decrease in the values of $y_{Max}$ and $y_{min}$ in a ECD case was observed, and this decrease tended to zero when the distance x increased. 

The resulting variations of the different geometric characteristics of the grain profile in the diffusion alone and in the simultaneous case of evaporation/condensation and diffusion are certainly due to the thermal effect of the evaporation/condensation phenomenon. A decrease in the surface area of the groove profile was observed in the combined cases, proving the important competition between evaporation and diffusion, as it was shown in Section 8.

**Figure 5:**
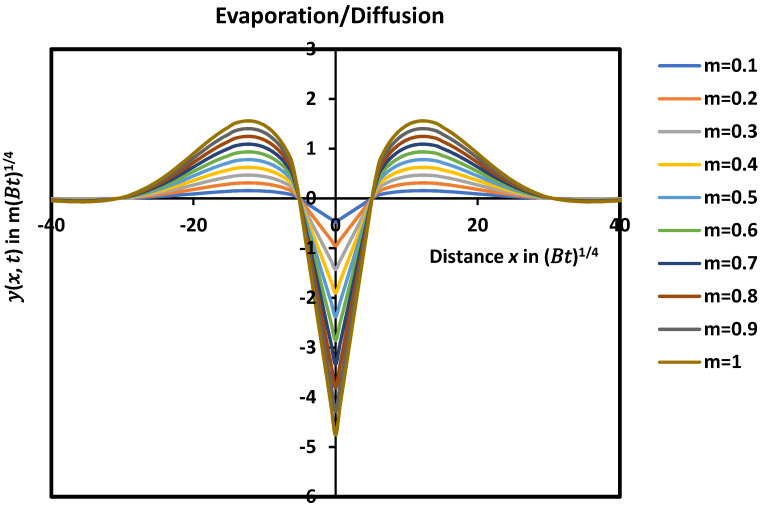
Evolution of the groove profile $y\left(x,\;t\right)$ as a function of the distance x in the case of simultaneous effects of evaporation/condensation and diffusion for different values of *m* corresponding to a contact angle of the groove from θ = 5.7° to 45°.

The effect of the slope m at the origin or the contact angle on the shape of the groove profile was studied and is shown in Figure 5. It was shown that the groove depth increased when the contact angle of the groove θ increased while the other characteristics, such as $d_{Max}$ and the width $w_{Max}$ of the groove, remained the same.

**Figure 6:**
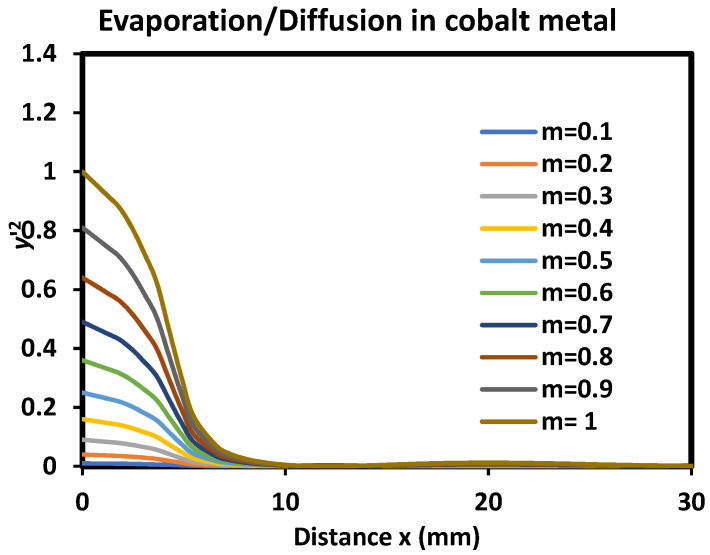
Variation of ${y\prime \left(x\right)}^{2}$ as a function of the distance x in the case of simultaneous effects of evaporation/condensation and diffusion for different values contact angle of the groove from θ = 5.7° to 45° (case of cobalt metal).

The analytical rigorous solution was obtained for ${y\prime }^{2}\ll 1$. The variations of ${y^{\prime }}^{2}(x)$ versus the distance *x* were plotted in Figure 6. Two conclusions can be deduced from the curves of Figure 6. The first one was for the validity of the above approximation; it was concluded that the solution remains still valid even for all values of ${y\prime }^{2}$ with the condition of contact angle of the groove less than 27.6°. Knowing that the contact angle was defined as the angle formed between the *y*-axis and the tangent at the origin of the groove, it can be deduced that the total contact angle of the groove can reach 54.2° without any loss on the analytical solution. The second conclusion was that the analytical solution remains valid for distances x≥6 μm even if ${y\prime }^{2}$ is not neglected behind 1.

## 7. Analytical Solution by Using the Series Development

Equation (20) can be also written as follows:

(30) $$g^{\prime \prime \prime \prime }-ag^{\prime \prime }-\frac{1}{4}\;ug^{\prime }+\frac{1}{4}g=0$$

By considering the development of g(u) in whole series as a function u, one writes the following:

(31) $$g\left(u\right)=\overset{\infty }{\underset{i=0}{\sum }}a_{i}u^{i}$$

The different derivatives of the above whole series are given by Equation (32):

$$ug\prime \left(u\right)=\sum_{i=0}^{\infty }{ia}_{i}u^{i}$$

$$g^{\prime \prime }\left(u\right)=\sum_{i=2}^{\infty }{i\left(i-1\right)a}_{i}u^{i-2}=\sum_{i=0}^{\infty }{(i+1)(i+2)a}_{i+2}u^{i}$$

$$g^{\prime \prime \prime \prime }\left(u\right)=\sum_{i=4}^{\infty }{i\left(i-1\right)\left(i-2\right)\left(i-3\right)a}_{i}u^{i-4}=\sum_{i=0}^{\infty }{(i+1)(i+2)(i+3)(i+4)a}_{i+4}u^{i}$$

Equation (30) can then be transformed into the following equation for all values of u by replacing all derivatives with their developments:

$$\overset{\infty }{\underset{i=0}{\sum }}\left[{(i+1)(i+2)(i+3)(i+4)a}_{i+4}-a{\left(i+1\right)\left(i+2\right)a}_{i+2}-\frac{1}{4}{ia}_{i}+\frac{1}{4}a_{i}\right]u^{i}=0,\;\forall u\in R$$

and one obtains a recurrent relation between the different terms $a_{i}$ of the series representing the function $g\left(u\right)$:

(32) $${(i+1)(i+2)(i+3)(i+4)a}_{i+4}-a{\left(i+1\right)\left(i+2\right)a}_{i+2}+\frac{1}{4}(1-i)a_{i}=0,\;\forall i\in N$$

The complete determination of coefficients $a_{i}$ can be obtained by knowing the first four terms of the series $a_{0}$, $a_{1}$, $a_{2}$, and $a_{3}$. The other terms $a_{i},\;\forall i\geq 4\;$can be deduced by recurrent relations. The boundary conditions of the grain grooving profile are used to determine the different terms of the series. Two types of boundary conditions can be applied: the first one was proposed by Mullins [14,15], and the second one by Amram et al. [64].

The use of the boundary conditions obtained by Mullins led to the following relations:

(33) $$\left\{\begin{array}{c} a_{0}=-\frac{1}{\sqrt{2}\times \Gamma \left(5/4\right)}\\ a_{1}=1\\ a_{2}=-\frac{1}{8\sqrt{2}\times \Gamma \left(\frac{5}{4}\right)}\\ a_{3}=0 \end{array}\right.$$

Using relation $a_{2}=\frac{a_{0}}{8}$ and Equation (33), one obtained the expression of $a_{4}$ as a function of the evaporation/diffusion coefficient a:

$$a_{4}=\frac{\left(a-1\right)\;a_{0}}{4!\times 4}=-\frac{\left(a-1\right)}{4!\times 4\sqrt{2}\times \Gamma \left(\frac{5}{4}\right)}$$

The calculations proved that the odd terms of the series are zero, and one writes:

(34) $${\;a}_{2n+1}=0\;\forall n\in N$$

The even terms are given by the following recurrent relation:

(35) $${\;a}_{2n}=\frac{4a{\left(2n-3\right)\left(2n-2\right)a}_{2n-2}-(2n-5)a_{2n-4}}{8n\left(2n-1\right)\left(2n-2\right)\left(2n-3\right)}=0,\;\forall n\in N$$

Therefore, the analytical solution $g\left(u\right)=\sum_{i=0}^{\infty }a_{i}u^{i}$ was fully determined and obviously convergent towards zero.

By using the boundary conditions proposed by Amram et al. [54], one writes Equation (36):

(36) $$\left\{\begin{array}{c} a_{0}=-\frac{\sqrt{2}}{\Gamma \left(5/4\right)}\;\\ a_{1}=1\\ a_{2}=0\\ a_{3}=-\frac{1}{6\sqrt{\pi }} \end{array}\right.$$

This allowed the provision of the value of $a_{4}=-\frac{a_{0}}{96}$ and the recurrent formula giving all values $a_{n}$:

(37) $${\;a}_{n+4}=\frac{4a{\left(n+1\right)\left(n+2\right)a}_{n+2}+(n-1)a_{n}}{4(n+1)\left(n+2\right)\left(n+3\right)\left(n+4\right)}=0,\;\forall n\in N$$

**Figure 7:**
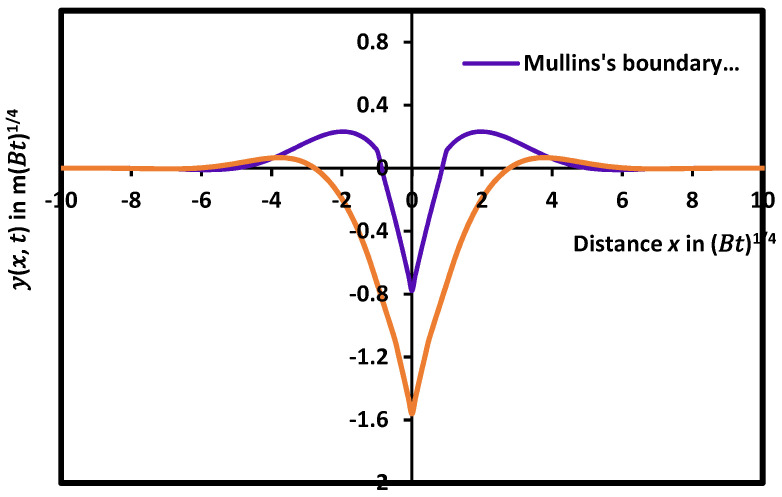
Dependence of the grain groove profile and its geometric characteristics on the boundary conditions in the case of evaporation/condensation and diffusion acting on polycrystalline thin films.

It was found that the boundary conditions proposed by Mullins [12,13] and Amram et al. [64] gave a very large difference between the two solutions. Figure 7 showed that the maximum of the groove with the boundary conditions of Amram et al. [64] was four times smaller than that obtained by using the boundary conditions of Mullins, whereas the grain groove deep was twice deeper than that of Mullins’s conditions. All geometric characteristics of the grain groove profile are affected by the change in the boundary conditions. It seems that the classical Mullins-type groove growing by surface diffusion alone cannot be satisfied by the presence of an evaporation/condensation process. The correction made by this present work represents a key microstructural element of thin metal polycrystalline films and, in many cases, participates in the determination of their physical, mechanical, and functional properties.

## 8. Evolution of the Profile Area in the General Case of Evaporation and Diffusion

The general partial differential equation governing the grain boundary grooving submitted to evaporation/condensation and diffusion was given by the following:

(38) $$\frac{\partial y}{\partial t}=C\frac{y^{\prime \prime }}{\left(1+y^{\prime 2}\right)}-B\;\frac{\partial }{\partial x}\left[{\left(1+{y\prime }^{2}\right)}^{-2}\;\left[y^{\prime \prime \prime }-3y\prime {y\prime \prime }^{2}{\left(1+{y\prime }^{2}\right)}^{-1}\right]\right]$$

The profile area of the groove ≙ was given by the following integral:

(39) $$\frac{d≙}{dt}=2\int_{0}^{+\infty }\left(\frac{\partial y}{\partial t}\right)dx$$

The integration of Equation (39) led to the following solution:

(40) $$\frac{d≙}{dt}=C\times Arctan\;m+\frac{3B^{1/2}m^{3}{\left(1+m^{2}\right)}^{-3}}{32\times \Gamma \left(\frac{5}{4}\right)}t^{-1/2}$$

Using the boundary conditions (41):

(41) $$\left\{\begin{array}{c} y^{\prime }\left(0\right)=m\\ y^{\prime \prime }\left(0\right)=-\frac{m{\left(Bt\right)}^{-1/4}}{4\sqrt{2}\times \Gamma \left(\frac{5}{4}\right)}\\ y^{\prime \prime \prime }\left(0\right)=0 \end{array}\right.$$

the integration of Equation (40), with respect to time t, led to Equation (42) giving the profile area of the grain boundary grooving as a function of time:

(42) $$≙=Ct\;Arctan\;m+\frac{3B^{1/2}m^{3}{\left(1+m^{2}\right)}^{-3}}{16\times \Gamma \left(\frac{5}{4}\right)}t^{1/2}$$

Equation (42) obtained from the general partial differential equation without any particular approximation is composed of two terms of the profile area relative to the evaporation/condensation and diffusion, respectively, given by the following relations:

(43) $$\left\{\begin{array}{c} {≙}_{E/C}=Ct\;Arctan\;m\\ {≙}_{D}=\frac{3B^{1/2}m^{3}{\left(1+m^{2}\right)}^{-3}}{16\times \Gamma \left(\frac{5}{4}\right)}t^{1/2} \end{array}\right.$$

Considering that m=tan θ, where θ is the contact angle of the groove at origin, relations (42) can be written as follows:

(44) $$\left\{\begin{array}{c} {≙}_{E/C}=Ct\;\theta \\ {≙}_{D}=\frac{3B^{1/2}\;{tan\;\theta \;}^{3}{\left(1+{tan\;\theta \;}^{2}\right)}^{-3}}{16\times \Gamma \left(\frac{5}{4}\right)}t^{1/2} \end{array}\right.$$

To study the variations of the different profile areas of several metals heavily used in power electronic devices, one took the experimental data from previous works [62,63] and gave the values of B and C in Table 3.

**Figure 8:**
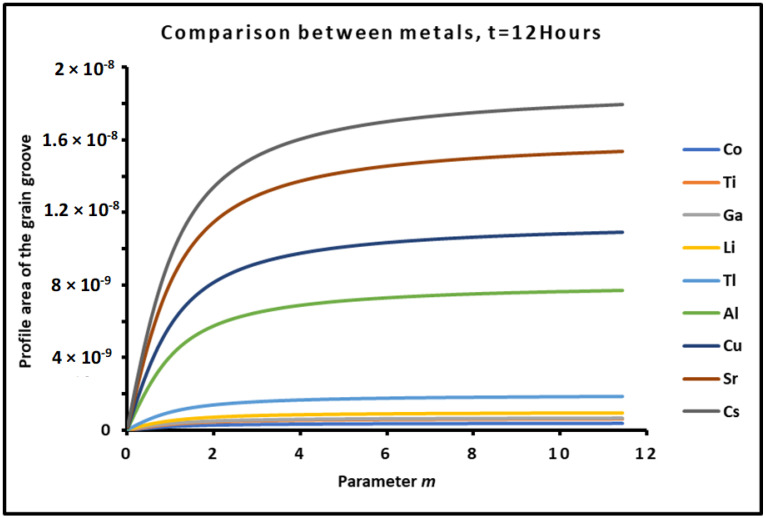
Evolution of the profile area of metals as a function of the parameter m=tan θ, for a fixed time t=12 h.

Experimental results allowed us to draw, in Figure 8, the variations of the profile area of metals as a function of the parameter m=tan θ, for a fixed time t=12 h. The curves of Figure 1 show an increasing profile area of metals when the contact angle θ increases. The comparison between the results of the different metals led to the classification of these metals in order of increasing profile area as follows:

*Co* < *Ti* < *Ga* < *Li* < *Tl* < *Al* < *Cu* < *Sr* < *Cs* (45)

The above classification (45) is in good agreement with the evaporation and diffusion constants. The results showed that when these constants increase, the profile area increases, too.

**Figure 9:**
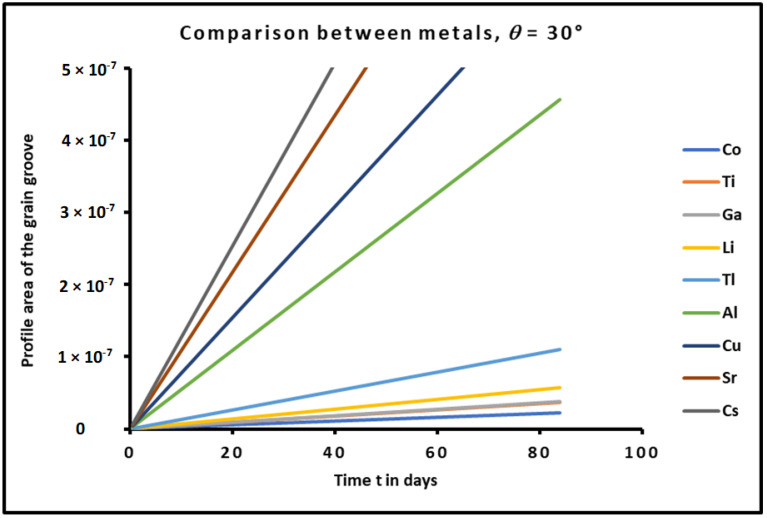
Variations of the profile area of metals as a function of the time *t* (in days) for a contact angle θ=30° or m=0.58.

The effect of the time on the profile of metals was shown in Figure 9 for a contact angle of the grain groove equal to θ=30° or for m=0.58. The results obviously confirmed the increase in the profile area when the time increased. This quantified the effect of time on the mechanical and thermal degradation of polycrystalline metals. The time effect gave the same classification order (given by relation (45)) of the profile area of the different metals.

**Figure 10:**
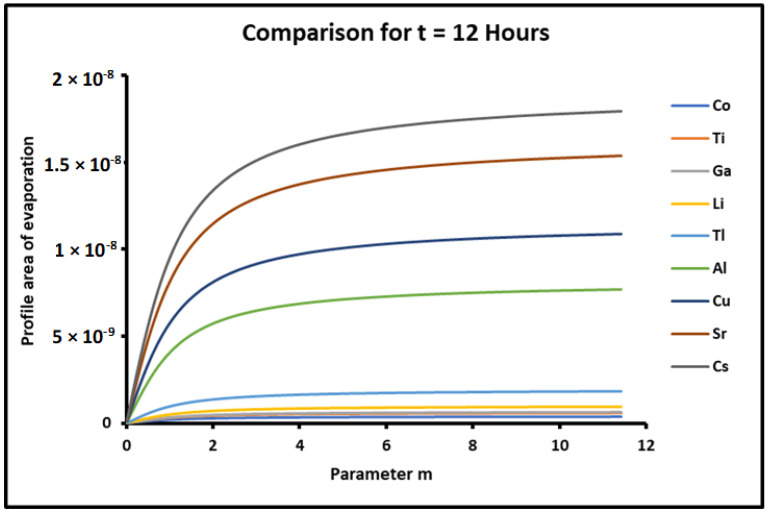
Evolution of the profile area of metals due to the evaporation as a function of the parameter m=tan θ, for a fixed time t=12 h.

**Figure 11:**
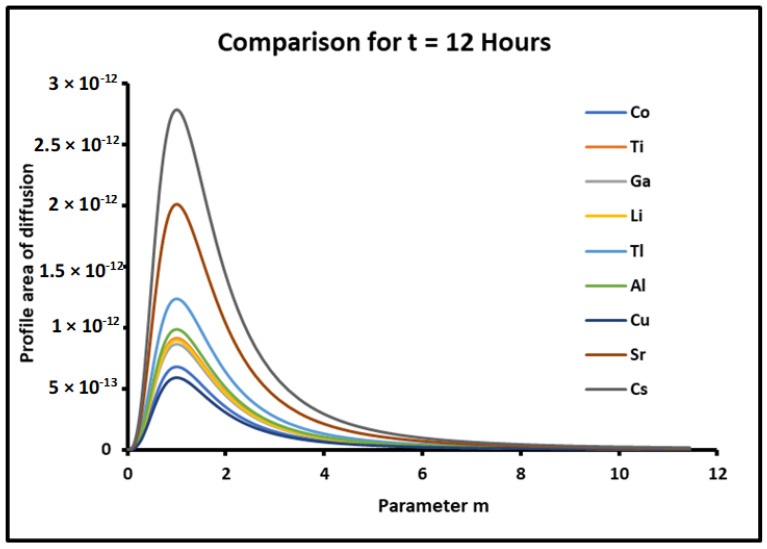
Evolution of the profile area of metals due to the diffusion as a function of the parameter m=tan θ, for a fixed time t=12 h.

The results relative to the two contributions of evaporation and diffusion in the values of the profile area of metals are represented in Figure 10 and Figure 11.

The results in Figure 10 showed similar variations of the profile area due to the evaporation process compared to those of Figure 8, and the same classification order of metals was obtained. However, the curves in Figure 11 relative to the diffusion part of the profile area presented non-monotonous variations. Indeed, the profile area due to the diffusion increased until a maximum of the parameter $m=m_{Max}=1$ corresponding to a contact angle θ=45° for all metals followed by a decrease for θ>45°. The metals were classified in increasing order of the profile area as follows:

*Cu* < *Co* < *Ga* < *Li* < *Ti* < *Al* < *Tl* < *Sr* < *Cs* (46)

Relation (46) gave an order of metals completely different from that of relation (45) except for metals Sr and Cs, which always exhibited the highest profile area.

**Figure 12:**
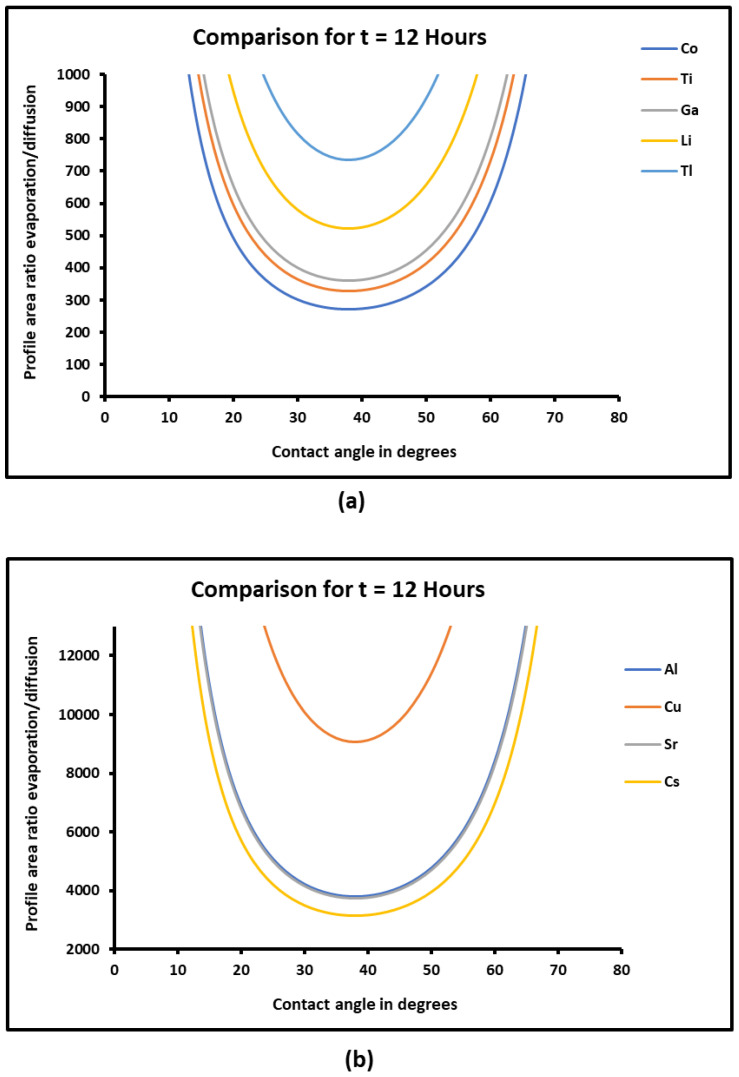
Evolution of the profile area ratio of evaporation/diffusion of metals (**a**): *Co*, *Ti*, *Ga*, *Li*, *Tl* and (**b**): *Al*, *Cu*, *Sr*, *Cs* as a function of the contact angle θ (in °), for a fixed time t=12 h.

Now, in order to compare the two effects of evaporation and diffusion, we drew in Figure 12 the evolution of the profile area ratio of evaporation/diffusion for t=12 h. 

Figure 12a,b show that the ratio of evaporation on diffusion for all metals decreased until a minimum of the contact angle equal to θ=38°. The results highlighted a higher effect of the diffusion for θ<38°, while the evaporation effect predominated for θ>38°. However, in all studied cases, it was shown that the ratio evaporation/diffusion is greater than 250 for all tested metals, proving that even if the diffusion exerted an important effect for θ<38°, the profile area of the evaporation was at least 250 times higher than that corresponding to the diffusion.

## 9. Conclusions

A new analytical solution to the partial differential equation $\frac{\partial y}{\partial t}=Cy^{\prime \prime }-By^{\prime \prime \prime \prime }$ was proposed in the combined cases of evaporation/condensation and diffusion in polycrystalline thin films. A detailed mathematical method was given. The determination of the groove profile $y\left(x,\;t\right)$ and its derivatives allowed us to conclude that the validity of the new mathematical solution can be extended to the domain located out of that relative to the approximation ${y\prime }^{2}\ll 1$. Indeed, the obtained solution is valid even if the contact angle of the groove is greater than 30° for distance *x* greater than 6 μm. It was shown that the values of the parameters $x_{Max\;}$, $x_{min}$, $∆x_{Max\;}$, and $x_{min}$ of the groove profile were practically the same in the case of diffusion alone and combined evaporation/diffusion case. However, an important decrease in the values of $y_{Max}$ and $y_{min}$ in the combined case was observed, proving the capital role of the evaporation on the characteristics of the grain groove profile. The accurate determination of the profile area relative to the evaporation, diffusion, and combined case led to the interesting conclusion of the highest effect of the evaporation relative to that of the diffusion. This conclusion was confirmed by the experimental data of several metals, thus showing the important effect of evaporation. The effects of the time and contact angle on the profile area were highlighted in different metals. A maximum of the profile area relative to the diffusion was obtained for a contact angle θ=45°, while a minimum in the ratio of the profile areas of evaporation on diffusion was observed for θ=38°. Furthermore, another analytical solution of the partial differential equation using the series development method was proposed. 

## Figures and Tables

**Table 1:** Values of the coordinates of maxima ($x_{Max};\;$$y_{Max}$) and minima ($x_{min};$ $y_{min}$) of the function y(x,t), the differences between two consecutive maxima ($∆x_{Max}$) and minima ($∆x_{min}$), and the zeros ($x_{0}$) of *y* for the combined effect of evaporation and diffusion and the alone diffusion case.

Grain Boundary Groove Parameters of the Combined Cases of Evaporation/Condensation and Diffusion							
Number N	$x_{Max}$ $\mathrm{in}\;{\left(Bt\right)}^{1/4\;}$	$y_{Max}$ $\mathrm{in}\;m{\left(Bt\right)}^{1/4\;}$	$∆x_{Max}$ $\mathrm{in}\;{\left(Bt\right)}^{1/4\;}$	$x_{min}$ $\mathrm{in}\;{\left(Bt\right)}^{1/4\;}$	$y_{min}$ $\mathrm{in}\;m{\left(Bt\right)}^{1/4\;}$	$∆x_{min}$ $\mathrm{in}\;{\left(Bt\right)}^{1/4\;}$	$x_{0}\;$ $\mathrm{in}\;{\left(Bt\right)}^{1/4\;}$ Zeros of y
1	2.02	2.56 × 10^−1^	-	5.88	−1.13 × 10^−2^	-	0.85
2	8.58	9.44 × 10^−4^	6.56	10.88	−9.20 × 10^−5^	5.00	5.06
3	12.98	9.69 × 10^−6^	4.41	15.08	−1.06 × 10^−6^	4.20	7.83
4	16.88	1.24 × 10^−7^	3.92	18.68	−1.23 × 10^−8^	3.60	10.23
5	20.52	1.27 × 10^−9^	3.64	22.13	−1.42 × 10^−10^	3.45	12.33
6	23.88	1.31 × 10^−11^	3.36	25.41	−1.64 × 10^−12^	3.28	14.43
7	27.08	1.34 × 10^−13^	3.22	28.56	−1.90 × 10^−14^	3.15	16.53
8	30.18	1.37 × 10^−15^	3.14	31.62	−2.20 × 10^−16^	3.06	18.63
**Grain Boundary Groove Parameters of the Alone Diffusion Case**							
1	2.4	2.60 × 10^−1^	-	5.22	−4.02 × 10^−2^	-	1.22
2	7.62	6.44 × 10^−3^	5.22	9.66	−1.05 × 10^−3^	4.44	4.35
3	11.62	1.70 × 10^−4^	4.00	13.7	−2.57 × 10^−5^	4.04	6.78
4	15.26	4.50 × 10^−6^	3.64	16.98	−7.33 × 10^−7^	3.28	9
5	18.62	1.19 × 10^−7^	3.36	20.26	−1.95 × 10^−8^	3.28	11
6	21.82	3.17 × 10^−9^	3.20	23.34	−5.17 × 10^−10^	3.08	12.89
7	24.82	8.42 × 10^−11^	3.00	26.3	−1.37 × 10^−11^	2.96	14.69
8	27.74	2.24 × 10^−12^	2.92	29.14	−3.64 × 10^−13^	2.84	16.44

**Table 2:** Ratios of the different parameters of the grain groove profile in the combined case (ECD) and the diffusion alone (D).

Number N	$\frac{x_{Max\left(ECD\right)}}{x_{Max(D)}}$	$\frac{y_{Max(ECD)}}{y_{Max(D)}}$	$\frac{{\Delta x}_{Max(ECD)}}{{\Delta x}_{Max(D)}}$	$\frac{x_{min(ECD)}}{x_{min(D)}}$	$\frac{y_{min(ECD)}}{y_{min(D)}}$	$\frac{{\Delta x}_{min(ECD)}}{{\Delta x}_{min(D)}}$	$\frac{x_{0(ECD)}}{x_{0(D)}}$
1	0.84	9.83 × 10^−1^	-	1.13	2.81 × 10^−1^	-	0.70
2	1.13	1.47 × 10^−1^	1.26	1.13	8.76 × 10^−2^	1.13	1.16
3	1.12	5.70 × 10^−2^	1.10	1.10	4.14 × 10^−2^	1.04	1.15
4	1.11	2.76 × 10^−2^	1.08	1.10	1.68 × 10^−2^	1.10	1.14
5	1.10	1.07 × 10^−2^	1.08	1.09	7.29 × 10^−3^	1.05	1.12
6	1.09	4.12 × 10^−3^	1.05	1.09	3.18 × 10^−3^	1.06	1.12
7	1.09	1.59 × 10^−3^	1.07	1.09	1.39 × 10^−3^	1.06	1.13
8	1.09	6.13 × 10^−4^	1.08	1.09	6.04 × 10^−4^	1.08	1.13

**Table 3:** Values of evaporation *C* and diffusion *B* constants of different metals.

Metal	$C\;\left(in\;m^{2}/s\right)$	$B\;\left(in\;m^{4}/s\right)$
*Co*	5.9 × 10^−15^	1.6 × 10^−26^
*Ti*	9.6 × 10^−15^	2.9 × 10^−26^
*Ga*	1.0 × 10^−14^	2.6 × 10^−26^
*Li*	1.5 × 10^−14^	2.8 × 10^−26^
*Tl*	2.9 × 10^−14^	5.3 × 10^−26^
*Al*	1.2 × 10^−13^	3.4 × 10^−26^
*Cu*	1.7 × 10^−13^	1.2 × 10^−26^
*Sr*	2.4 × 10^−13^	1.4 × 10^−25^
*Cs*	2.8 × 10^−13^	2.7 × 10^−25^

## Data Availability

The original contributions presented in the study are included in the article, further inquiries can be directed to the corresponding authors.

## Appendix A

To resolve Equation (6), we used the same method developed in a previous study [53] by transforming Equation (6) into the difference between two perfect squares and then transforming the expression ${\left(8\mu +4a\right)r}^{2}+u\;r+{4\mu }^{2}-1$ into a perfect square; it has to have a double solution, and then its discriminant has to be canceled.

Now, considering the following equation:

(A1) $${\left(8\mu +4a\right)r}^{2}+u\;r+{4\mu }^{2}-1=0$$

and cancelling the discriminant ∆ of this second-degree equation function in r, one obtains

(A2) $$∆=u^{2}-16\;\left(2\mu +a\right)\left(4\mu^{2}-1\right)$$

Putting ∆=0, one has

(A3) $$\mu^{3}+\frac{a}{2}\mu^{2}-\frac{1}{4}\mu -\frac{u^{2}+16a}{128}=0$$

By choosing $\mu =\eta -\frac{a}{6}$, one obtains

(A4) $$\eta^{3}-\frac{a^{2}+3}{12}\eta +\frac{32\;a\;\left(a^{3}-9\right)-27\;u^{2}}{128*27}=0$$

Equation (A4) can be written as general form:

(A5) $$\eta^{3}+p\eta +q=0$$

With $p=-\frac{a^{2}+3}{12}$ and $q=+\frac{32\;\left(a^{3}-9a\right)-27\;u^{2}}{128*27}$.

Putting η=α+β and taking $\alpha \beta =-\frac{p}{3}$ or $\alpha^{3}\beta^{3}=-\frac{p^{3}}{27}$, one obtains:

$$\alpha^{3}+\beta^{3}=-q$$

And $\alpha^{3}$ and $\beta^{3}$ will be the solutions of the following second-degree Equation (A6):

(A6) $$X^{2}+qX-\frac{p^{3}}{27}=0$$

With the following discriminant:

(A7) $${∆}_{\eta }=\frac{{{27q}^{2}+4p}^{3}}{27}={\left[\frac{32\;\left(a^{3}-9a\right)-27\;u^{2}}{2^{7}*3^{3}}\right]}^{2}-{\frac{4}{27}\left[\frac{a^{2}+3}{2^{2}*3}\right]}^{3}$$

or

(A8) $${∆}_{\eta }=\frac{{{27q}^{2}+4p}^{3}}{27}=\frac{{\left[32\;\left(a^{3}-9a\right)-27\;u^{2}\right]}^{2}-{2^{10}\left(a^{2}+3\right)}^{3}}{2^{14}*3^{6}}$$

(A9) $$\left\{\begin{array}{c} {∆}_{\eta }=\;\frac{{\left(a_{1}-u^{2}\right)}^{2}-a_{2}^{2}}{2^{14}}\\ a_{1}=\frac{2^{5}\;\left(a^{3}-9a\right)}{3^{3}};\;\left(a^{3}-9a\right)>0\\ a_{2}^{2}={\frac{2^{10}}{3^{6}}\left(a^{2}+3\right)}^{3} \end{array}\right.$$

Therefore

$$q=\frac{a_{1}-u^{2}}{128}$$

$$p=-\frac{a^{2}+3}{12}$$

Two cases have to be considered:

(A10) $$\left\{\begin{array}{c} {∆}_{\eta }>0⟺u>\sqrt{a_{1}+a_{2}}\\ {∆}_{\eta }<0⟺u<\sqrt{a_{1}+a_{2}} \end{array}\right.$$

The limit value of *u* is given by

(A11) $$u_{0}=\sqrt{a_{1}+a_{2}}$$

1. First case ${∆}_{\eta }>0$ for u $>u_{0}$

In this case, one obtains

$$\alpha^{3}=\frac{-q+\sqrt{{∆}_{\eta }}}{2}\;\mathrm{and}\;\beta^{3}=\frac{-q-\sqrt{{∆}_{\eta }}}{2}$$

One writes

(A12) $$\left\{\begin{array}{c} \alpha^{3}=\frac{-\left(a_{1}-u^{2}\right)+\sqrt{{\left(a_{1}-u^{2}\right)}^{2}-a_{2}^{2}}}{2^{8}}\\ \beta^{3}=\frac{-\left(a_{1}-u^{2}\right)-\sqrt{{\left(a_{1}-u^{2}\right)}^{2}-a_{2}^{2}}}{2^{8}} \end{array}\right.$$

This leads to the solution of Equation (A5):

(A13) η=α+β

And this also leads to the solution of Equation (A6):

(A14) $$\mu =\alpha +\beta -\frac{a}{6}$$

This value of *μ* will cancel the discriminant of Equation (A1). The solution *r* is then given by

(A15) $$r=-\frac{u}{8\;\left(2\mu +a\right)}$$

Therefore

(A16) $${\left(8\mu +4a\right)r}^{2}+u\;r+{4\mu }^{2}-1=4\left(2\mu +a\right){\left(r+\frac{u}{8\;\left(2\mu +a\right)}\right)}^{2}$$

Knowing that $\left(2\mu +a\right)<0$, one obtains

(A17) $${{E\left(r\right)=r}^{4}-a{\;r}^{2}-\frac{1}{4}u\;r+\frac{1}{4}=\left(r^{2}+\mu \right)}^{2}-\left(2\mu +a\right){\left(r+\frac{u}{8\;\left(2\mu +a\right)}\right)}^{2}$$

And in complex development, one obtains

(A18) $$E\left(r\right)=\left(r^{2}+\mu -i\;\sqrt{\left(-2\mu -a\right)}\;\left(r+\frac{u}{8\sqrt{\left(2\mu +a\right)}}\right)\right)\left(r^{2}+\mu +i\;\sqrt{\left(-2\mu -a\right)}\;\left(r+\frac{u}{8\sqrt{\left(2\mu +a\right)}}\right)\right)$$

One deduced that, in this case, the four solutions will be complex. The solutions of Equation (A18) are equivalent to the solutions of the two following equations:

(A19) $$r^{2}-i\;\sqrt{\left(-2\mu -a\right)}\;r+\mu -i\frac{u\sqrt{\left(-2\mu -a\right)}}{8\left(2\mu +a\right)}=0$$

(A20) $$r^{2}+i\;\sqrt{\left(-2\mu -a\right)}\;r+\mu +i\frac{u\sqrt{\left(-2\mu -a\right)}}{8\left(2\mu +a\right)}=0$$

The discriminants of Equations (A19) and (A20) are given by the respective following expressions:

(A21) $${∆}_{1}=\left(a-2\mu \right)+i\frac{u\sqrt{\left(-2\mu -a\right)}}{2\left(2\mu +a\right)}$$

(A22) $${∆}_{2}=\left(a-2\mu \right)-i\frac{u\sqrt{\left(-2\mu -a\right)}}{2\left(2\mu +a\right)}$$

The two discriminants can be given as

(A23) $$\left\{\begin{array}{c} {∆}_{1}=\rho \;e^{i\theta }\;\;\;\\ {∆}_{2}=\rho \;e^{-i\theta } \end{array}\right.$$

The cases studied here are obtained for $u>u_{0}$. The four complex solutions are then given by

(A24) $$\left\{\begin{array}{c} r_{1}=\frac{\rho^{1/2}\;con\left(\frac{\theta }{2}\right)+i\left[\sqrt{\left(-2\mu -a\right)}+sin\left(\frac{\theta }{2}\right)\right]}{2}\\ r_{2}=\frac{{-\rho }^{1/2}\;con\left(\frac{\theta }{2}\right)+i\left[\sqrt{\left(-2\mu -a\right)}-sin\left(\frac{\theta }{2}\right)\right]}{2}\\ r_{3}=\frac{\rho^{1/2}\;con\left(\frac{\theta }{2}\right)-i\left[\sqrt{\left(-2\mu -a\right)}+sin\left(\frac{\theta }{2}\right)\right]}{2}\\ r_{4}=\frac{{-\rho }^{1/2}\;con\left(\frac{\theta }{2}\right)-i\left[\sqrt{\left(-2\mu -a\right)}-sin\left(\frac{\theta }{2}\right)\right]}{2} \end{array}\right.$$

Now, the solution in this case for $u>u_{0}$, is given by

$$g_{1}\left(u\right)=Exp\left(-\rho^{\frac{1}{2}}\;con\left(\frac{\theta }{2}\right)\right)\left[A_{11}cos\left(\left(\sqrt{\left(-2\mu -a\right)}-sin\left(\frac{\theta }{2}\right)\right)\;u\right)+A_{21}sin\left(\left(\sqrt{\left(-2\mu -a\right)}-sin\left(\frac{\theta }{2}\right)\right)\;u\right)\right]$$

(A25) $$+Exp\left(\rho^{1/2}\;con\left(\frac{\theta }{2}\right)u\right)\left[A_{31}cos\left(\left(\sqrt{\left(-2\mu -a\right)}+sin\left(\frac{\theta }{2}\right)\right)\;u\right)+A_{41}sin\left(\left(\sqrt{\left(-2\mu -a\right)}+sin\left(\frac{\theta }{2}\right)\right)\;u\right)\right]$$

The following are the boundary conditions:

(A26) $$\left\{\begin{array}{c} 1)\;\underset{u\to \infty }{\lim}g\left(u\right)=0\\ 2)\;\;\underset{u\to \infty }{\lim}g^{\prime }\left(u\right)=0\\ 3)\underset{u\to \infty }{\lim}g^{\prime \prime }\left(u\right)=0\;\;\;\;\;\;\;\;\;\;\;\;\;\;\;\;\;\;\;\;\;\; \end{array}\right.$$

This allows the deduction of the following parameters:

$$A_{31}=A_{41}=0$$

And the final solution is, therefore, given by

(A27) $$g_{1}\left(u\right)=Exp\left[\left(-\rho^{1/2}\;cos\left(\frac{\theta }{2}\right)\right)u\right]\left[A_{11}cos\left[\left(\sqrt{\left(-2\mu -a\right)}-sin\left(\frac{\theta }{2}\right)\right)\;u\right]+A_{21}sin\left[\left(\sqrt{\left(-2\mu -a\right)}-sin\left(\frac{\theta }{2}\right)\right)\;u\right]\right]$$

where

(A28) $$\left\{\begin{array}{c} \rho =\sqrt{{\left(a-2\mu \right)}^{2}-\frac{u^{2}}{2\;(a+2\mu )}}\\ \rho^{1/2}\;con\left(\frac{\theta }{2}\right)=\sqrt{\frac{\rho +a-2\mu }{2}}\\ sin\left(\frac{\theta }{2}\right)=\sqrt{\frac{\rho -a+2\mu }{2\;\rho }} \end{array}\right.$$

Equation (A27) can be written in general form as

(A29) $$g_{1}\left(u\right)=e^{-p_{1}(u)}\;\left(A_{11}cos\;q_{1}(u)+A_{21}sin\;q_{1}(u)\right)$$

With

(A30) $$\left\{\begin{array}{c} p_{1}\left(u\right)=\sqrt{\frac{\rho +a-2\mu }{2}}u\\ q_{1}\left(u\right)=\left[\sqrt{\left(-2\mu -a\right)}-\sqrt{\frac{\rho -a+2\mu }{2\;\rho }}\right]u \end{array}\right.$$

where $a_{2}^{2}$, $\alpha^{3}$, $\beta^{3}$, and μ are, respectively, given by Equations (A9), (A12), and (A14).

The three first derivatives of the function $g_{1}\left(u\right)$ are given by the following equations:

$${g_{1}}^{\prime }\left(u\right)={-p_{1}}^{\prime }\;\left(A_{11}\cos q_{1}+A_{21}\sin q_{1}\right)e^{-p_{1}}+q_{1}\prime \;\left(A_{21}\cos q_{1}-A_{11}\sin q_{1}\right)e^{-p_{1}}\;{g_{1}}^{\prime \prime }\left(u\right)=\left({p_{1}\prime }^{2}-{q_{1}\prime }^{2}-{p_{1}}^{\prime \prime }\right)\left(A_{11}\cos q_{1}+A_{21}\sin q_{1}\right)e^{-p_{1}}+\left({q_{1}}^{\prime \prime }\;{-2\;p_{1}}^{\prime }q_{1}\prime \right)\;\left(A_{21}\cos q_{1}-A_{11}\sin q_{1}\right)e^{-p_{1}}\;{g_{1}}^{\prime \prime \prime }\left(u\right)=\left(3{p_{1}}^{\prime }p^{\prime \prime }-3{q_{1}}^{\prime }{q_{1}}^{\prime \prime }-{p_{1}}^{\prime \prime \prime }{-{p_{1}}^{\prime }}^{3}\;{+3\;p_{1}}^{\prime }{q_{1}\prime }^{2}\right)\left(A_{11}\cos q_{1}+A_{21}\sin q_{1}\right)e^{-p_{1}}\;+\left({3p_{1}\prime }^{2}{q_{1}}^{\prime }-{{q_{1}}^{\prime }}^{3}-3{p_{1}}^{\prime \prime }{q_{1}}^{\prime }+{q_{1}}^{\prime \prime \prime }-3{p_{1}}^{\prime }q_{1}\prime \prime \right)\left(A_{21}\cos q_{1}-A_{11}\sin q_{1}\right)e^{-p_{1}}$$

2. Second case ${∆}_{\eta }<0$ for $u<u_{0}$

In this case, one obtains

(A31) $$\left\{\begin{array}{c} \alpha^{3}=\rho \;e^{i\omega }\;\;\;\\ \beta^{3}=\rho \;e^{-i\omega } \end{array}\right.$$

where $\rho^{2}={\left|\alpha^{3}\right|}^{2}={\left|\beta^{3}\right|}^{2}=\frac{a_{2}^{2}}{2^{16}}=\frac{{\left(a^{2}+3\right)}^{3}}{2^{6}{\times 3}^{6}}$, and $\rho =\frac{{\left(a^{2}+3\right)}^{3/2}}{2^{3}{\times 3}^{3}}$

and finally, $\rho^{1/3}=\frac{\sqrt{\left(a^{2}+3\right)}}{6}$

With

(A32) $$\left\{\begin{array}{c} \eta =\frac{\sqrt{\left(a^{2}+3\right)}}{3}cos\left(\frac{\omega }{3}\right)={2\;\rho }^{1/3}cos\left(\frac{\omega }{3}\right)\\ \rho \;cos\;\omega =\frac{-\left(a_{1}-u^{2}\right)}{2^{8}}\;;\;\rho =\frac{{\left(a^{2}+3\right)}^{3/2}}{2^{3}{\times 3}^{3}}\\ \rho \;sin\;\omega =\frac{\sqrt{{a_{2}^{2}-\left(a_{1}-u^{2}\right)}^{2}}}{2^{8}} \end{array}\right.$$

And the solution of Equation (A1) is as follows:

(A33) $$\mu =\eta -\frac{a}{6}$$

This value of *μ* will cancel the discriminant of Equation (A1). The solution *r* is given by

$$r=-\frac{u}{8\;\left(2\mu +a\right)}$$

The expression $E\left(r\right)$ can be given by

(A34) $$E\left(r\right)=\left(r^{2}+\sqrt{\left(2\mu +a\right)}\;r+\mu +\frac{u}{8\sqrt{\left(2\mu +a\right)}}\right)\left(r^{2}-\sqrt{\left(2\mu +a\right)}\;r+\mu -\frac{u}{8\sqrt{\left(2\mu +a\right)}}\right)$$

And, therefore

(A35) $$\left\{\begin{array}{c} \left(r^{2}+\sqrt{\left(2\mu +a\right)}\;r+\mu +\frac{u}{8\sqrt{\left(2\mu +a\right)}}\right)=0\\ \left(r^{2}-\sqrt{\left(2\mu +a\right)}\;r+\mu -\frac{u}{8\sqrt{\left(2\mu +a\right)}}\right)=0 \end{array}\right.$$

The discriminants of Equation (A35) are given by the respective following expressions:

(A36) $$\left\{\begin{array}{c} {∆}_{1}=\left(a-2\mu \right)-\frac{u}{2\sqrt{\left(a+2\mu \right)}}\\ {∆}_{2}=\left(a-2\mu \right)+\frac{u}{2\sqrt{\left(a+2\mu \right)}} \end{array}\right.$$

In this case, one has

$$\mu =\eta -\frac{a}{6}=\frac{\sqrt{\left(a^{2}+3\right)}}{3}cos\left(\frac{\omega }{3}\right)-\frac{a}{6}$$

$${\mu =2\;\rho }^{1/3}cos\left(\frac{\omega }{3}\right)-\frac{a}{6}$$

Two cases can be studied:

First case: $r^{2}+\sqrt{\left(2\mu +a\right)}\;r+\mu +\frac{u}{8\sqrt{\left(2\mu +a\right)}}=0$

where ${∆}_{1}=\left(a-2\mu \right)-\frac{u}{2\sqrt{\left(a+2\mu \right)}}\;$ is positive for $u<u_{0}$

(A37) $$r_{1}=\frac{1}{2}\left[-\sqrt{\left(2\mu +a\right)}+\sqrt{\left(a-2\mu \right)-\frac{u}{2\sqrt{\left(a+2\mu \right)}}}\right]$$

(A38) $$r_{2}=\frac{1}{2}\left[-\sqrt{\left(2\mu +a\right)}-\sqrt{\left(a-2\mu \right)-\frac{u}{2\sqrt{\left(a+2\mu \right)}}}\right]$$

Second case: $\left(r^{2}-\sqrt{\left(2\mu +a\right)}\;r+\mu -\frac{u}{8\sqrt{\left(2\mu +a\right)}}\right)=0$

where ${∆}_{2}=\left(a-2\mu \right)+\frac{u}{2\sqrt{\left(a+2\mu \right)}}$ is positive for $u<u_{0}$

The two other solutions are then given by

(A39) $$r_{3}=\frac{1}{2}\left[\sqrt{\left(2\mu +a\right)}+\sqrt{\left(a-2\mu \right)+\frac{u}{2\sqrt{\left(a+2\mu \right)}}}\right]$$

(A40) $$r_{4}=\frac{1}{2}\left[\sqrt{\left(2\mu +a\right)}-\sqrt{\left(a-2\mu \right)+\frac{u}{2\sqrt{\left(a+2\mu \right)}}}\right]$$

Now, the final solution for $u<u_{0}$, is given by

(A41) $$g_{2}\left(u\right)=e^{-\frac{1}{2}\times \sqrt{\left(2\mu +a\right)}\;u}\left(A_{12}Exp\left(\frac{1}{2}\times \sqrt{\left(a-2\mu \right)-\frac{u}{2\sqrt{\left(a+2\mu \right)}}}\;u\right)+A_{22}Exp\left(-\frac{1}{2}\times \sqrt{\left(a-2\mu \right)-\frac{u}{2\sqrt{\left(a+2\mu \right)}}}\;u\right)\right)\;+e^{\frac{1}{2}\times \sqrt{\left(2\mu +a\right)}\;u}\left(A_{32}Exp\left(\frac{1}{2}\times \sqrt{\left(a-2\mu \right)+\frac{u}{2\sqrt{\left(a+2\mu \right)}}}\;u\right)+A_{42}Exp\left(-\frac{1}{2}\times \sqrt{\left(a-2\mu \right)+\frac{u}{2\sqrt{\left(a+2\mu \right)}}}\;u\right)\right)$$

Putting

$$l\left(u\right)=\frac{1}{2}\sqrt{\left(2\mu (u)+a\right)};\;\;\;p\left(u\right)=l(u)\;u\;q_{1}\left(u\right)=\frac{1}{2}\times \sqrt{\left(a-2\mu \right)-\frac{u}{2\sqrt{\left(a+2\mu \right)}}}\;u\;\mathrm{or}\;q_{1}\left(u\right)=\frac{1}{2}\times \sqrt{\left(a-2\mu \right)-\frac{u{\left(l\left(u\right)\right)}^{-1}}{4}}\;u\;q_{2}\left(u\right)=\frac{1}{2}\times \sqrt{\left(a-2\mu \right)+\frac{u}{2\sqrt{\left(a+2\mu \right)}}}\;u\;\mathrm{or}\;q_{2}\left(u\right)=\sqrt{\left(a-2\mu \right)+\frac{u{\left(l\left(u\right)\right)}^{-1}}{4}}\;u$$

One obtains, for $u<u_{0}$, the following solution $g_{2}\left(u\right)$:

$$g_{2}\left(u\right)=e^{-p(u)}\left(A_{12}e^{q_{1}\left(u\right)}+A_{22}e^{{-q}_{1}\left(u\right)}\right)+e^{p(u)}\left(A_{32}e^{q_{2}\left(u\right)}+A_{42}e^{{-q}_{2}\left(u\right)}\right)$$

In conclusion, for $u>u_{0}$

$$g_{1}\left(u\right)=Exp\left(-\rho^{1/2}\;con\left(\frac{\theta }{2}\right)\right)\left[A_{11}cos\left(\left(\sqrt{\left(-2\mu -a\right)}-sin\left(\frac{\theta }{2}\right)\right)\;u\right)+A_{21}sin\left(\left(\sqrt{\left(-2\mu -a\right)}-sin\left(\frac{\theta }{2}\right)\right)\;u\right)\right]\;g_{1}\left(u\right)=e^{-p_{1}(u)}\;\left(A_{11}cos\;q_{1}(u)+A_{21}sin\;q_{1}(u)\right)$$

With

$$p_{1}\left(u\right)=\left(-\rho^{1/2}\;con\left(\frac{\theta }{2}\right)\right)u;\;q_{1}\left(u\right)=\left(\sqrt{\left(-2\mu -a\right)}-sin\left(\frac{\theta }{2}\right)\right)\;u$$

$$l_{1}\left(u\right)={\left[-2\mu \left(u\right)-a\right]}^{1/2};\;\mu =\eta -\frac{a}{6};\;\eta =\alpha +\beta \;\;\;\;\;\;\;\;\;\;\;\;\;\;\;\;\;\;\;\;\;\;\;\;\;\alpha^{3}=\frac{-\left(a_{1}-u^{2}\right)+\sqrt{{\left(a_{1}-u^{2}\right)}^{2}-a_{2}^{2}}}{2^{8}}\;\;\;\;\;\;\;\;\;\;\;\;\;\;\;\;\;\;\;\;\;\;\;\;\;\beta^{3}=\frac{-\left(a_{1}-u^{2}\right)-\sqrt{{\left(a_{1}-u^{2}\right)}^{2}-a_{2}^{2}}}{2^{8}}$$

And for $u<u_{0}$

$$g_{2}\left(u\right)=e^{-p(u)}\left(A_{12}e^{q_{1}\left(u\right)}+A_{22}e^{{-q}_{1}\left(u\right)}\right)+e^{p(u)}\left(A_{32}e^{q_{2}\left(u\right)}+A_{42}e^{{-q}_{2}\left(u\right)}\right)\;l\left(u\right)=\frac{1}{2}\sqrt{\left(2\mu (u)+a\right)};\;\;\;p\left(u\right)=l(u)\;u\;q_{1}\left(u\right)=\frac{1}{2}\times \sqrt{\left(a-2\mu \right)-\frac{u}{2\sqrt{\left(a+2\mu \right)}}}\;u\;\mathrm{or}\;q_{1}\left(u\right)=\frac{1}{2}\times \sqrt{\left(a-2\mu \right)-\frac{u{\left(l\left(u\right)\right)}^{-1}}{4}}\;u\;q_{2}\left(u\right)=\frac{1}{2}\times \sqrt{\left(a-2\mu \right)+\frac{u}{2\sqrt{\left(a+2\mu \right)}}}\;u\;\mathrm{or}\;q_{2}\left(u\right)=\sqrt{\left(a-2\mu \right)+\frac{u{\left(l\left(u\right)\right)}^{-1}}{4}}\;u\;l\left(u\right)=\frac{1}{2}\sqrt{\left(2\mu (u)+a\right)};\;p\left(u\right)=\frac{1}{2}\sqrt{\left(2\mu +a\right)}\;u=ul(u);\;\mu =\eta -\frac{a}{6}$$

The three first derivatives of $g_{2}\left(u\right)$ are given below:

$${g\prime }_{2}={e^{-p}\left[\left(-p^{\prime }+{q\prime }_{1}\right)\;A_{12}e^{q_{1}}-\left(p^{\prime }+{q\prime }_{1}\right)A_{22}e^{{-q}_{1}}\right]+e}^{p}\left[\left(p^{\prime }+{q\prime }_{2}\right)A_{32}e^{q_{2}}+\left(p\prime {-q\prime }_{2}\right)A_{42}e^{{-q}_{2}}\right]\;{g\prime \prime }_{2}={e^{-p}\left[\left[{\left(p^{\prime }-{q\prime }_{1}\right)}^{2}-p^{\prime \prime }+{q\prime \prime }_{1}\right]\;A_{12}e^{q_{1}}+\left[{\left(p^{\prime }+{q\prime }_{1}\right)}^{2}-p^{\prime \prime }-{q\prime \prime }_{1}\right]A_{22}e^{{-q}_{1}}\right]+e}^{p}\left[\left[{\left(p^{\prime }+{q\prime }_{2}\right)}^{2}+p^{\prime \prime }+{q\prime \prime }_{2}\right]\;A_{32}e^{q_{2}}+\left[{\left(p^{\prime }-{q\prime }_{2}\right)}^{2}+p^{\prime \prime }-{q\prime \prime }_{2}\right]A_{42}e^{{-q}_{2}}\right]\;{g\prime \prime \prime }_{2}={e^{-p}\left[\left[{\left(-p^{\prime }+{q\prime }_{1}\right)}^{3}+3p\prime p^{\prime \prime }-3p\prime {q^{\prime \prime }}_{1}-3p\prime \prime {q^{\prime }}_{1}+3{q^{\prime }}_{1}{q^{\prime \prime }}_{1}-p\prime \prime \prime +{q\prime \prime \prime }_{1}\right]\;A_{12}e^{q_{1}}+\left[{\left(-p^{\prime }-{q\prime }_{1}\right)}^{3}+3p^{\prime p^{\prime \prime }}+3p\prime {q^{\prime \prime }}_{1}+3p\prime \prime {q^{\prime }}_{1}+3{q^{\prime }}_{1}{q^{\prime \prime }}_{1}-p\prime \prime \prime -{q\prime \prime \prime }_{1}\right]A_{22}e^{{-q}_{1}}\right]+e}^{p}\left[\left[{\left(p^{\prime }+{q\prime }_{2}\right)}^{3}+3p\prime p^{\prime \prime }+3p\prime {q^{\prime \prime }}_{2}+3p\prime \prime {q^{\prime }}_{2}+3{q^{\prime }}_{2}{q^{\prime \prime }}_{2}+p\prime \prime \prime +{q\prime \prime \prime }_{2}\right]\;A_{32}e^{q_{2}}+\left[{\left(p^{\prime }-{q\prime }_{2}\right)}^{3}+3p\prime p^{\prime \prime }-3p\prime {q^{\prime \prime }}_{2}-3p\prime \prime {q^{\prime }}_{2}+3{q^{\prime }}_{2}{q^{\prime \prime }}_{2}+p\prime \prime \prime -{q\prime \prime \prime }_{2}\right]A_{42}e^{{-q}_{2}}\right]$$

And the function g and its different derivatives are given by the following boundary conditions at point 0:

$$\left\{\begin{array}{c} g\left(0\right)=-\frac{1}{\sqrt{2}\times \Gamma \left(5/4\right)}\\ g^{\prime }\left(0\right)=1\\ g^{\prime \prime }\left(0\right)=-\frac{1}{4\sqrt{2}\times \Gamma \left(\frac{5}{4}\right)}=\frac{g\left(0\right)}{4}\\ g^{\prime \prime \prime }\left(0\right)=0 \end{array}\right.$$

The expressions of the function $g_{2}$ and derivatives in 0 are given below as a function of the different parameters *Aij*

$$g_{2}\left(0\right)=A_{12}+A_{22}+A_{32}+A_{42}=g_{0}=-\frac{1}{\sqrt{2}\Gamma \left(5/4\right)}\;{g^{\prime }}_{2}\left(0\right)=\left(-p^{\prime }+{q^{\prime }}_{1}\right)\;A_{12}-\left(p^{\prime }+{q^{\prime }}_{1}\right)A_{22}+\left(p^{\prime }+{q^{\prime }}_{2}\right)A_{32}+\left(p^{\prime {-q^{\prime }}_{2}}\right)A_{42}=1\;{g^{\prime \prime }}_{2}\left(0\right)=\left[{\left(p^{\prime }-{q^{\prime }}_{1}\right)}^{2}-p^{\prime \prime }+{q^{\prime \prime }}_{1}\right]\;A_{12}+\left[{\left(p^{\prime }+{q^{\prime }}_{1}\right)}^{2}-p^{\prime \prime }-{q^{\prime \prime }}_{1}\right]A_{22}\;+\left[{\left(p^{\prime }+{q\prime }_{2}\right)}^{2}+p^{\prime \prime }+{q\prime \prime }_{2}\right]\;A_{32}+\left[{\left(p^{\prime }-{q\prime }_{2}\right)}^{2}+p^{\prime \prime }-{q\prime \prime }_{2}\right]A_{42}=\frac{g\left(0\right)}{4}\;{g^{\prime \prime \prime }}_{2}\left(0\right)=\left[{\left(-p^{\prime }+{q^{\prime }}_{1}\right)}^{3}+3p^{\prime }p^{\prime \prime }-3p^{\prime }{q^{\prime \prime }}_{1}-3p^{\prime \prime }{q^{\prime }}_{1}+3{q^{\prime }}_{1}{q^{\prime \prime }}_{1}-p^{\prime \prime \prime }+{q^{\prime \prime \prime }}_{1}\right]\;A_{12}+\left[{\left(-p^{\prime }-{q^{\prime }}_{1}\right)}^{3}+3p^{\prime }p^{\prime \prime }+3p^{\prime }{q^{\prime \prime }}_{1}+3p^{\prime \prime }{q^{\prime }}_{1}+3{q^{\prime }}_{1}{q^{\prime \prime }}_{1}-p^{\prime \prime \prime }-{q^{\prime \prime \prime }}_{1}\right]A_{22}+\left[{\left(p^{\prime }+{q^{\prime }}_{2}\right)}^{3}+3p^{\prime }p^{\prime \prime }+3p^{\prime }{q^{\prime \prime }}_{2}+3p^{\prime \prime }{q^{\prime }}_{2}+3{q^{\prime }}_{2}{q^{\prime \prime }}_{2}+p^{\prime \prime \prime }+{q^{\prime \prime \prime }}_{2}\right]\;A_{32}+\left[{\left(p^{\prime }-{q^{\prime }}_{2}\right)}^{3}+3p^{\prime }p^{\prime \prime }-3p^{\prime }{q^{\prime \prime }}_{2}-3p^{\prime \prime }{q^{\prime }}_{2}+3{q^{\prime }}_{2}{q^{\prime \prime }}_{2}+p^{\prime \prime \prime }-{q^{\prime \prime \prime }}_{2}\right]A_{42}=0$$

Taking

$p\left(u\right)=\frac{1}{2}\sqrt{\left(2\mu (u)+a\right)}\;u$; $l\left(u\right)=\frac{1}{2}\sqrt{\left(2\mu (u)+a\right)}$ and $p\left(u\right)=l(u)\;u$; one obtains the different derivatives of l(u) and $p\left(u\right)$:

$$l^{\prime }=\frac{1}{2}\frac{\mu^{\prime }}{\sqrt{\left(2\mu +a\right)}};\;l\prime \prime =\frac{1}{2}\left[\mu \prime \prime {\left(2\mu +a\right)}^{-1/2}-{\mu \prime }^{2}{\left(2\mu +a\right)}^{-3/2}\right]\;l\prime \prime \prime =\frac{1}{2}\left[\mu \prime \prime \prime {\left(2\mu +a\right)}^{-\frac{1}{2}}-3\mu^{\prime }\mu^{\prime \prime }{\left(2\mu +a\right)}^{-\frac{1}{2}}+3{\mu \prime }^{3}{\left(2\mu +a\right)}^{-5/2}\right]\;p\prime =l+l\prime \;u\;;\;p\prime \prime =2l\prime +l\prime \prime \;u;\;p\prime \prime \prime =3l\prime \prime +l\prime \prime \prime \;u$$

Putting

$q_{1}\left(u\right)=\frac{1}{2}\sqrt{\left(a-2\mu \right)-\frac{u}{2\sqrt{\left(a+2\mu \right)}}}\;u$ or $q_{1}\left(u\right)=\frac{1}{2}\sqrt{\left(a-2\mu \right)-\frac{u{\left(l\left(u\right)\right)}^{-1}}{4}}\;u$; $q_{1}\left(u\right)=\frac{1}{2}\sqrt{m_{1}(u)}u$; $m_{1}(u)=\left(a-2\mu \right)-\frac{u}{2\sqrt{\left(a+2\mu \right)}}=\left(a-2\mu \right)-\frac{ul^{-1}}{4}$

One obtains the derivatives of $m_{1}(u)$ and $q_{1}\left(u\right)$:

$$\left\{\begin{array}{c} {m_{1}}^{\prime }=\frac{1}{4}\;\left[-l^{-1}+u\;l\prime l^{-2}\right]-2\mu \prime \;\;\;\\ {m_{1}}^{\prime \prime }=\frac{1}{4}\;\left[2l\prime l^{-2}+l\prime \prime l^{-2}u-2{l\prime }^{2}l^{-3}u\right]-2\mu \prime \prime \;\;\\ {m_{1}}^{\prime \prime \prime }=\frac{1}{4}\;\left[u\;l\prime \prime \prime l^{-2}-6l\prime l\prime \prime l^{-3}u-6{l\prime }^{2}l^{-3}+6{l\prime }^{3}l^{-4}u+3l\prime \prime l^{-2}\right]-2\mu \prime \prime \prime \;\; \end{array}\right.\;\left\{\begin{array}{c} {q_{1}}^{\prime }=\frac{1}{2}\left[\frac{1}{2}\;{m_{1}}^{\prime }{m_{1}}^{-\frac{1}{2}}u+{m_{1}}^{1/2}\right]\;\;\;\;\;\\ {q_{1}}^{\prime \prime }=\frac{1}{2}\left[\frac{1}{2}\;{m_{1}}^{\prime \prime }{m_{1}}^{-\frac{1}{2}}u-\frac{1}{4}\;{{m_{1}}^{\prime }}^{2}{m_{1}}^{-\frac{3}{2}}u+{m_{1}}^{\prime }{m_{1}}^{-\frac{1}{2}}\right]\;\;\\ {q_{1}}^{\prime \prime \prime }=\frac{1}{2}\left[\frac{1}{2}\;{m_{1}}^{\prime \prime \prime }{m_{1}}^{-\frac{1}{2}}u-\frac{3}{4}\;{{m_{1}}^{\prime }m_{1}}^{\prime \prime }{m_{1}}^{-\frac{3}{2}}u+\frac{3}{8}\;{{m_{1}}^{\prime }}^{3}{m_{1}}^{-\frac{5}{2}}u+\frac{3}{2}\;{m_{1}}^{\prime \prime }{m_{1}}^{-\frac{1}{2}}-\frac{3}{4}\;{{m_{1}}^{\prime }}^{2}{m_{1}}^{-\frac{3}{2}}\right]\;\; \end{array}\right.$$

The same procedure was used with $m_{2}(u)$ and $q_{2}\left(u\right)$:

$q_{2}\left(u\right)=\frac{1}{2}\sqrt{\left(a-2\mu \right)+\frac{u}{2\sqrt{\left(a+2\mu \right)}}}\;u$ or $q_{2}\left(u\right)=\frac{1}{2}\sqrt{\left(a-2\mu \right)+\frac{u{\left(l\left(u\right)\right)}^{-1}}{4}}\;u$; $q_{2}\left(u\right)=\frac{1}{2}\sqrt{m_{2}(u)}u$; $m_{2}=\left(a-2\mu \right)+\frac{ul^{-1}}{4}$

$$\left\{\begin{array}{c} {m_{2}}^{\prime }=-\frac{1}{4}\;\left[-l^{-1}+u\;l\prime l^{-2}\right]-2\mu \prime \;\;\;\\ {m_{2}}^{\prime \prime }=-\frac{1}{4}\;\left[2l\prime l^{-2}+l\prime \prime l^{-2}u-2{l\prime }^{2}l^{-3}u\right]-2\mu \prime \prime \;\;\\ {m_{2}}^{\prime \prime \prime }=-\frac{1}{4}\;\left[u\;l\prime \prime \prime l^{-2}-6l\prime l\prime \prime l^{-3}u-6{l\prime }^{2}l^{-3}+6{l\prime }^{3}l^{-4}u+3l\prime \prime l^{-2}\right]-2\mu \prime \prime \prime \;\; \end{array}\right.\;q_{2}\left(u\right)=\frac{1}{2}\sqrt{m_{2}(u)}u\;\left\{\begin{array}{c} {q_{2}}^{\prime }=\frac{1}{2}\left[\frac{1}{2}\;{m_{2}}^{\prime }{m_{2}}^{-\frac{1}{2}}u+{m_{2}}^{1/2}\right]\;\;\;\;\;\\ {q_{2}}^{\prime \prime }=\frac{1}{2}\left[\frac{1}{2}\;{m_{2}}^{\prime \prime }{m_{2}}^{-\frac{1}{2}}u-\frac{1}{4}\;{{m_{2}}^{\prime }}^{2}{m_{2}}^{-\frac{3}{2}}u+{m_{2}}^{\prime }{m_{2}}^{-\frac{1}{2}}\right]\;\;\\ {q_{2}}^{\prime \prime \prime }=\frac{1}{2}\left[\frac{1}{2}\;{m_{2}}^{\prime \prime \prime }{m_{2}}^{-\frac{1}{2}}u-\frac{3}{4}\;{{m_{2}}^{\prime }m_{2}}^{\prime \prime }{m_{2}}^{-\frac{3}{2}}u+\frac{3}{8}\;{{m_{2}}^{\prime }}^{3}{m_{2}}^{-\frac{5}{2}}u+\frac{3}{2}\;{m_{2}}^{\prime \prime }{m_{2}}^{-\frac{1}{2}}-\frac{3}{4}\;{{m_{2}}^{\prime }}^{2}{m_{2}}^{-\frac{3}{2}}\right]\;\; \end{array}\right.$$

Values in 0 for $q_{1}\left(u\right)$ and all derivatives

$$l\left(0\right)=\frac{1}{2}\sqrt{\left(2\mu (0)+a\right)};\;p\left(0\right)=0\;;\;q\left(0\right)=0\;;\;m\left(0\right)=\left(a-2\mu (0)\right)\;\mu \left(0\right)={2\;\rho }^{1/3}cos\left(\frac{\omega (0)}{3}\right)-\frac{a}{6}\;\left\{\begin{array}{c} \mu \prime \left(0\right)=0\;\;\;\;\;\;\\ \mu \prime \prime \left(0\right)=\frac{1}{{2^{6}\rho }^{\frac{2}{3}}}\left(\frac{sin\left(\frac{\omega }{3}\right)}{sin\;\omega }\right)(0)\;\;\;\;\;\;\;\\ \mu \prime \prime \prime \left(0\right)=0\;\;\;\;\;\;\;\;\;\;\;\;\;\; \end{array}\right.\;l\left(0\right)=\frac{1}{2}\sqrt{\left(2\mu (0)+a\right)}\;\left\{\begin{array}{c} l\prime \left(0\right)=0\;\;\;\;\;\;\\ l^{\prime \prime }(0)=\frac{1}{2}\mu \prime \prime (0){\left(2\mu (0)+a\right)}^{-1/2}\;\;\;\;\;\;\;\\ l\prime \prime \prime \left(0\right)=0\;\; \end{array}\right.\;p\left(0\right)=0\;;\;p\prime \left(0\right)=l(0)\;;\;p\prime \prime \left(0\right)=0\;;\;p\prime \prime \prime \left(0\right)=3l\prime \prime (0)\;m_{1}\left(0\right)=\left(a-2\mu (0)\right)\;\left\{\begin{array}{c} {m_{1}}^{\prime }\left(0\right)=-\frac{1}{4}\;\left[{l\left(0\right)}^{-1}\right]\\ {m_{1}}^{\prime \prime }\left(0\right)={-2\mu }^{\prime \prime }\left(0\right)=\frac{-2}{{2^{6}\rho }^{\frac{2}{3}}}\left(\frac{sin\left(\frac{\omega }{3}\right)}{sin\;\omega }\right)(0)\;\;\mathrm{well-verified}\\ {m_{1}}^{\prime \prime \prime }\left(u\right)=\frac{3}{4}\;l\prime \prime \left(0\right){l\left(0\right)}^{-2}\;\;\; \end{array}\right.\;q_{1}\left(0\right)=0\;\left\{\begin{array}{c} {q_{1}}^{\prime }\left(0\right)={\frac{1}{2}m_{1}(0)}^{1/2}\;\;\;\;\;\\ {q_{1}}^{\prime \prime }\left(0\right)=\frac{1}{2}{m_{1}}^{\prime }\left(0\right){m_{1}\left(0\right)}^{-\frac{1}{2}}\;\;\\ {q_{1}}^{\prime \prime \prime }\left(u\right)=\frac{1}{2}\left[\frac{3}{2}\;{m_{1}}^{\prime \prime }\left(0\right){m_{1}\left(0\right)}^{-\frac{1}{2}}-\frac{3}{4}\;{{m_{1}}^{\prime }\left(0\right)}^{2}{m_{1}\left(0\right)}^{-\frac{3}{2}}\right]\;\; \end{array}\right.$$

Values of different parameters at point 0 and derivatives

$$l\left(0\right)=\frac{1}{2}\sqrt{\left(2\mu (0)+a\right)}\;;\;p\left(0\right)=0\;;\;q\left(0\right)=0\;;\;m\left(0\right)=\left(a-2\mu (0)\right)\;\mu \left(0\right)={2\;\rho }^{1/3}cos\left(\frac{\omega (0)}{3}\right)-\frac{a}{6}\;\left\{\begin{array}{c} \mu \prime \left(0\right)=0\;\;\;\;\;\;\\ \mu \prime \prime \left(0\right)=\frac{1}{{2^{6}\rho }^{\frac{2}{3}}}\left(\frac{sin\left(\frac{\omega }{3}\right)}{sin\;\omega }\right)(0)\;\;\;\;\;\;\;\\ \mu \prime \prime \prime \left(0\right)=0\;\;\;\;\;\;\;\;\;\;\;\;\;\; \end{array}\right.\;l\left(0\right)=\frac{1}{2}\sqrt{\left(2\mu (0)+a\right)}\;\left\{\begin{array}{c} l\prime \left(0\right)=0\;\;\;\;\;\;\\ l^{\prime \prime }(0)=\frac{1}{2}\mu \prime \prime (0){\left(2\mu (0)+a\right)}^{-1/2}\;\;\;\;\;\;\;\\ l\prime \prime \prime \left(0\right)=0\;\; \end{array}\right.\;p\left(0\right)=0\;p\prime \left(0\right)=l(0)\;;\;p\prime \prime \left(0\right)=0\;;\;p\prime \prime \prime \left(0\right)=3l\prime \prime (0)\;m_{2}\left(0\right)=\left(a-2\mu (0)\right)\;\left\{\begin{array}{c} {m_{2}}^{\prime }\left(0\right)=\frac{1}{4}\;\left[{l\left(0\right)}^{-1}\right]\\ {m_{2}}^{\prime \prime }\left(0\right)={-2\mu }^{\prime \prime }\left(0\right)=\frac{-2}{{2^{6}\rho }^{\frac{2}{3}}}\left(\frac{sin\left(\frac{\omega }{3}\right)}{sin\;\omega }\right)(0)\;\;\\ {m_{2}}^{\prime \prime \prime }\left(u\right)=-\frac{3}{4}\;l\prime \prime \left(0\right){l\left(0\right)}^{-2}\;\;\; \end{array}\right.\;q_{2}\left(0\right)=0\;\left\{\begin{array}{c} {q_{2}}^{\prime }\left(0\right)=\frac{1}{2}{m_{2}(0)}^{1/2}\;\;\;\;\;\\ {q_{2}}^{\prime \prime }\left(0\right)=\frac{1}{2}{m_{2}}^{\prime }\left(0\right){m_{2}\left(0\right)}^{-\frac{1}{2}}\;\;\\ {q_{2}}^{\prime \prime \prime }\left(0\right)=\frac{1}{2}\left[\frac{3}{2}\;{m_{2}}^{\prime \prime }\left(0\right){m_{2}\left(0\right)}^{-\frac{1}{2}}-\frac{3}{4}\;{{m_{2}}^{\prime }\left(0\right)}^{2}{m_{2}\left(0\right)}^{-\frac{3}{2}}\right]\;\; \end{array}\right.$$

Comparison between parameters in 0

$$\left\{\begin{array}{c} m_{2}\left(0\right)=m_{1}(0)\\ {m_{2}}^{\prime }\left(0\right)=-m_{1}\prime (0)\\ {m_{2}}^{\prime \prime }\left(0\right)=m_{1}\prime \prime (0)\;\;\\ {m_{2}}^{\prime \prime \prime }\left(u\right)=-m_{1}\prime \prime \prime (0)\;\;\; \end{array}\right.\left\{\begin{array}{c} q_{2}\left(0\right)=q_{1}\left(0\right)\\ {q_{2}}^{\prime }\left(0\right)={q_{1}}^{\prime }\left(0\right)\;\;\;\;\;\\ {q_{2}}^{\prime \prime }\left(0\right)={-q_{1}}^{\prime \prime }\left(0\right)\\ {q_{2}}^{\prime \prime \prime }\left(0\right)={q_{1}}^{\prime \prime \prime }\left(0\right)\;\; \end{array}\right.$$

Calculation of the different derivatives of μ

Knowing that $\eta ={2\rho }^{1/3}cos\left(\frac{\omega }{3}\right)$; $cos\omega =\frac{-\left(a_{1}^{2}-u^{2}\right)}{2^{8}\rho }$ and $sin\omega =\frac{\sqrt{{b_{1}^{2}-\left(a_{1}^{2}-u^{2}\right)}^{2}}}{2^{8}\rho }$, one obtains $\eta^{’}\left(u\right)=\frac{d\eta }{du}=\frac{d\eta }{d\omega }.\frac{d\omega }{du}$; $\frac{d\omega }{du}=-{2\rho }^{1/3}sin\left(\frac{\omega }{3}\right)$; $\frac{d\omega }{du}=-\frac{2^{-7}}{\rho sin\omega }u$ and then the first derivative $\eta^{’}\left(u\right)$:

$${\mu^{\prime }\left(u\right)=\eta }^{\prime }\left(u\right)=\frac{1}{{2^{6}\rho }^{2/3}}\frac{sin\left(\frac{\omega }{3}\right)}{sin\;\omega }\;u$$

$\mu^{’}\left(u\right)=\frac{1}{{2^{6}\rho }^{2/3}}\frac{sin\left(\frac{\omega }{3}\right)}{sin\omega }u$; let us put $v\left(\omega \right)=\left(\frac{sin\left(\frac{\omega }{3}\right)}{sin\omega }\right)$; therefore, $\mu^{’’}\left(u\right)=\frac{1}{{2^{6}\rho }^{2/3}}\left(v+u\frac{dv}{du}\right)$

$$\mu^{\prime \prime }\left(u\right)=\frac{1}{{2^{6}\rho }^{2/3}}\;\left(v+u\;\frac{dv}{du}\;\right)\;;\;\frac{dv}{du}=\frac{dv}{d\omega }\;\frac{d\omega }{du}=\left[\frac{d}{d\omega }\left(\frac{sin\left(\frac{\omega }{3}\right)}{sin\;\omega }\right)\right]\times \left(\frac{-2^{-7}}{\rho \;sin\;\omega }\right)u$$

Knowing that $\frac{d}{d\omega }\left(\frac{sin\left(\frac{\omega }{3}\right)}{sin\omega }\right)=3^{-1}\frac{sin\omega cos\left(\frac{\omega }{3}\right)-3cos\omega sin\left(\frac{\omega }{3}\right)}{{sin}^{2}\omega }$, one obtains

$$\frac{dv}{du}=3^{-1}\frac{sin\;\omega \;cos\left(\frac{\omega }{3}\right)-3cos\;\omega \;sin\left(\frac{\omega }{3}\right)}{{sin}^{2}\;\omega }\times \left(\frac{-2^{-7}}{\rho \;sin\;\omega }\right)u\;\mu^{\prime \prime }\left(u\right)=\frac{1}{{2^{6}\rho }^{2/3}}\;\left[\left(\frac{sin\left(\frac{\omega }{3}\right)}{sin\;\omega }\right)+3^{-1}\frac{sin\;\omega \;cos\left(\frac{\omega }{3}\right)-3cos\;\omega \;sin\left(\frac{\omega }{3}\right)}{{sin}^{2}\;\omega }\times \left(\frac{-2^{-7}}{\rho \;sin\;\omega }\right)u^{2}\;\right]\;\;\mu^{\prime \prime }\left(u\right)=\frac{1}{{2^{6}\rho }^{2/3}}\;\left[\left(\frac{sin\left(\frac{\omega }{3}\right)}{sin\;\omega }\right)-\frac{u^{2}}{{3\times 2}^{7}\rho }\frac{sin\;\omega \;cos\left(\frac{\omega }{3}\right)-3cos\;\omega \;sin\left(\frac{\omega }{3}\right)}{{sin}^{3}\;\omega }\;\right]\;$$

By using $sinacosb=\frac{\sin\left(a+b\right)+\sin\left(a-b\right)}{2}$, one obtains

$$\frac{sin\;\omega \;cos\left(\frac{\omega }{3}\right)-3\;sin\left(\frac{\omega }{3}\right)\;cos\;\theta }{{sin}^{3}\;\omega }=\frac{2sin\left(\frac{2\omega }{3}\right)-sin\left(\frac{4\omega }{3}\right)\;}{{sin}^{3}\;\omega }$$

and, therefore

$$\mu^{\prime \prime }\left(u\right)=\frac{1}{{2^{6}\rho }^{2/3}}\;\left[\left(\frac{sin\left(\frac{\omega }{3}\right)}{sin\;\omega }\right)-\frac{u^{2}}{{3\times 2}^{7}\rho }\frac{2sin\left(\frac{2\omega }{3}\right)-sin\left(\frac{4\omega }{3}\right)\;}{{sin}^{3}\;\omega }\;\right]\;\mu^{\prime \prime \prime }\left(u\right)=\frac{1}{{2^{6}\rho }^{2/3}}\;\left[\frac{dv}{du}+\frac{u}{{3\times 2}^{6}\rho }\frac{2sin\left(\frac{2\omega }{3}\right)-sin\left(\frac{4\omega }{3}\right)\;}{{sin}^{3}\;\omega }+\frac{u^{2}}{{3\times 2}^{7}\rho }\frac{d}{d\omega }\left[\frac{2sin\left(\frac{2\omega }{3}\right)-sin\left(\frac{4\omega }{3}\right)\;}{{sin}^{3}\;\omega }\right]\times \left(-\frac{2^{-7}}{\rho \;sin\;\omega }u\right)\;\right]$$

Using $\frac{dv}{du}=-\frac{u^{2}}{{3\times 2}^{7}\rho }\frac{2sin\left(\frac{2\omega }{3}\right)-sin\left(\frac{4\omega }{3}\right)}{{sin}^{3}\omega }$, one obtains

$$\mu^{\prime \prime \prime }\left(u\right)=\frac{1}{{2^{6}\rho }^{2/3}}\;\left[-\frac{u^{2}}{{3\times 2}^{7}\rho }\frac{2sin\left(\frac{2\omega }{3}\right)-sin\left(\frac{4\omega }{3}\right)\;}{{sin}^{3}\;\omega }+\frac{u}{{3\times 2}^{6}\rho }\frac{2sin\left(\frac{2\omega }{3}\right)-sin\left(\frac{4\omega }{3}\right)\;}{{sin}^{3}\;\omega }-\frac{u^{3}}{{3\times 2}^{14}\rho^{2}sin\;\omega }\frac{d}{d\omega }\left[\frac{2sin\left(\frac{2\omega }{3}\right)-sin\left(\frac{4\omega }{3}\right)\;}{{sin}^{3}\;\omega }\right]\;\right]$$

With

$$\frac{d}{d\omega }\left[\frac{2sin\left(\frac{2\omega }{3}\right)-sin\left(\frac{4\omega }{3}\right)\;}{{sin}^{3}\;\omega }\right]=\frac{1}{6}\times \frac{35\;\;sin\left(\frac{\omega }{3}\right)-14\;sin\left(\frac{5\omega }{3}\right)+5\;sin\left(\frac{7\omega }{3}\right)\;}{{sin}^{4}\;\omega }$$

One writes

$$\mu^{\prime \prime \prime }\left(u\right)=\frac{1}{{2^{6}\rho }^{2/3}}\;\left[\frac{u}{{3\times 2}^{6}\rho }\frac{2sin\left(\frac{2\omega }{3}\right)-sin\left(\frac{4\omega }{3}\right)\;}{{sin}^{3}\;\omega }-\frac{u^{2}}{{3\times 2}^{7}\rho }\frac{2sin\left(\frac{2\omega }{3}\right)-sin\left(\frac{4\omega }{3}\right)\;}{{sin}^{3}\;\omega }-\frac{u^{3}}{{3^{2}\times 2}^{15}\rho^{2}}\times \frac{35\;\;sin\left(\frac{\omega }{3}\right)-14\;sin\left(\frac{5\omega }{3}\right)+5\;sin\left(\frac{7\omega }{3}\right)\;}{{sin}^{5}\;\omega }\;\right]$$

Values of the successive derivatives of μ in u = 0

$$\mu \left(0\right)={2\;\rho }^{1/3}cos\left(\frac{\omega (0)}{3}\right)-\frac{a}{6}\;\mu^{\prime }\left(u\right)=0\;\mu^{\prime \prime }\left(u\right)=\frac{1}{{2^{6}\rho }^{\frac{2}{3}}}\left(\frac{sin\left(\frac{\omega }{3}\right)}{sin\;\omega }\right)(0)\;\mu^{\prime \prime \prime }\left(u\right)=0$$
